# Mitochondrial-targeted actions of lycopene: evidence, mechanisms and future directions

**DOI:** 10.3389/fphar.2026.1818572

**Published:** 2026-06-25

**Authors:** Marcos Roberto de Oliveira

**Affiliations:** 1 Grupo de Estudos em Terapia Mitocondrial, Instituto de Ciências Básicas da Saúde (ICBS), Universidade Federal do Rio Grande do Sul (UFRGS), Porto Alegre, Rio Grande do Sul, Brazil; 2 Programa de Pós-Graduação em Alimentação, Nutrição e Saúde (PPGANS), Faculdade de Medicina, Universidade Federal do Rio Grande do Sul (UFRGS), Porto Alegre, Rio Grande do Sul, Brazil; 3 Grupo de Estudos em Neuroquímica e Neurobiologia de Moléculas Bioativas, Departamento de Química, Universidade Federal de Mato Grosso (UFMT), Cuiaba, Mato Grosso, Brazil

**Keywords:** apoptosis, LYCOPENE, mitochondria, mitochondrial biogenesis, mitochondrial function, mitophagy

## Abstract

Lycopene (LYC; C_40_H_56_), a dietary carotenoid, has emerged as a promising modulator of mitochondrial physiology across multiple cell types and animal models. Here we critically synthesize experimental evidence that LYC attenuates mitochondrial oxidative stress, preserves oxidative phosphorylation complex function and ATP production, reduces mitochondrial permeability transition and cytochrome-c–dependent apoptosis, and regulates mitochondrial quality-control pathways including mitophagy and (less consistently) biogenesis. Mechanistic readouts indicate activation of antioxidant axes (Nrf2/HO-1), modulation of SIRT1/SIRT3 and PGC-1 signaling, and downstream effects on Bcl-2 family proteins and caspase activation; targeted delivery systems (mitochondria-directed nanodots) further enhance mitochondrial targeting and functional rescue in neurodegeneration models. However, the literature shows substantial heterogeneity in experimental designs (dose, route, timing), mostly relies on injury/toxin paradigms, and frequently reports molecular changes without causal perturbation (genetic or pharmacologic) to establish mechanism. Importantly, data on mitochondrial dynamics (fusion/fission) remain sparse and mechanistic links between mitophagy, biogenesis and improved bioenergetics are often associative rather than causal. The objective of this review is to evaluate available evidence on how LYC modulates mitochondrial function, redox biology, biogenesis, dynamics, and autophagy (mitophagy), as well as mitochondria-dependent apoptosis, in animal and human cells, identify critical gaps, and propose experimental priorities to move the field toward translational studies. This work is concluded with concrete recommendations for mechanistic and translational research to validate LYC as a mitochondria-targeting agent.

## 1 Introduction

Mitochondrial dysfunction is increasingly recognized as a central feature of chronic pathological conditions, including neurodegenerative disorders, metabolic diseases, cardiovascular abnormalities, inflammatory syndromes, and cancer, in which impaired bioenergetics, oxidative disequilibrium, defective mitochondrial quality control, and dysregulated cell death pathways collectively contribute to disease progression (Prasun, 2020; Pollicino et al., 2023; Zong et al., 2024). Beyond their canonical role in adenosine triphosphate (ATP) synthesis, mitochondria function as dynamic signaling hubs integrating metabolic, inflammatory, calcium-dependent, and redox-responsive pathways that ultimately determine cellular adaptation and survival (Marchi et al., 2023). Consequently, compounds capable of modulating mitochondrial physiology have attracted increasing interest as potential therapeutic agents targeting disorders associated with mitochondrial dysfunction (de Oliveira, 2018; Infantino et al., 2024).

Among naturally occurring bioactive molecules, lycopene (LYC; C_40_H_56_) has emerged as one of the most intensively investigated carotenoids because of its potent antioxidant activity and broad biological effects (Arballo et al., 2021; Shafe et al., 2024). LYC is a highly lipophilic acyclic carotenoid predominantly found in tomatoes and tomato-derived products, although relevant amounts are also present in watermelon, papaya, guava, and pink grapefruit (Arballo et al., 2021). Structurally, LYC contains an extended system of conjugated double bonds that confers exceptional singlet oxygen-quenching capacity and facilitates interaction with reactive oxygen species (ROS), membrane lipids, and hydrophobic cellular compartments (Tufail et al., 2024). Importantly, this physicochemical configuration not only underlies its antioxidant potential but also critically influences its extraction efficiency, intracellular distribution, metabolic fate, and biological activity.

Because LYC is highly susceptible to oxidation, heat-induced degradation, and isomerization, extraction and preservation strategies substantially influence its downstream pharmacological behavior (Guerra et al., 2021). Conventional solvent-based extraction methods remain widely employed; however, emerging approaches involving high-pressure homogenization, microfluidization, and food-grade oil systems improve extraction efficiency while limiting oxidative degradation (Guerra et al., 2021). More recently, hydrophobic natural deep eutectic solvents composed of terpenes and fatty acids have been proposed as environmentally sustainable alternatives capable of achieving extraction efficiencies comparable to those obtained with traditional organic solvents (Kyriakoudi et al., 2022). These physicochemical considerations are highly relevant because the intrinsic instability of LYC directly affects its bioavailability, tissue distribution, and translational applicability.

Following oral administration, LYC is absorbed through mechanisms shared with dietary lipids, involving incorporation into mixed micelles, uptake by enterocytes, and systemic transport predominantly via circulating lipoproteins (Paul et al., 2020; Arballo et al., 2021). Nevertheless, intestinal absorption efficiency remains relatively low, generally ranging from approximately 7%–10% of total intake, and is strongly influenced by dietary fat composition, food matrix organization, and isomeric configuration (Paul et al., 2020). Cis-isomers exhibit greater bioavailability than the all-trans form because of enhanced solubility and reduced crystallinity (Ross et al., 2011). Under physiological conditions, circulating plasma concentrations typically remain within the low micromolar range (0.01–1.8 μmol/L) (Liu et al., 2006), an observation of major translational importance given that many experimental studies employ concentrations substantially exceeding physiologically achievable levels. Thus, a central unresolved issue in the field is whether the mitochondrial effects attributed to LYC occur at biologically relevant intracellular concentrations.

After absorption, LYC distributes selectively across tissues, accumulating preferentially in the liver, adrenal glands, testes, and prostate (Liu et al., 2006). Importantly, intracellular distribution appears to be highly context dependent. In hepatocytes, LYC exhibits approximately 3 - 5-fold enrichment within mitochondrial fractions relative to total tissue levels, suggesting preferential mitochondrial localization *in vivo* (Bradley et al., 2023). Although quantitative sub-mitochondrial distribution and molecular binding targets remain undefined, this observation provides a potentially important mechanistic basis for the mitochondrial effects associated with LYC. In contrast, studies in prostate cancer cells demonstrated predominant localization within nuclear-associated membrane structures rather than cytosolic compartments, reinforcing the concept that organelle-level exposure to LYC may vary substantially according to cellular phenotype, lipid composition, and metabolic context (Liu et al., 2006).

Metabolically, LYC undergoes extensive enzymatic cleavage primarily mediated by β-carotene oxygenases, particularly β-carotene oxygenase 2, generating multiple oxidized derivatives collectively termed lycopenoids (Arballo et al., 2021; Bradley et al., 2023). Although the biological activity of these metabolites remains incompletely characterized, increasing evidence suggests that at least part of the transcriptional and mitochondrial effects attributed to LYC may involve downstream metabolites rather than the parent molecule itself. Human tracer studies further demonstrate rapid post-absorptive metabolism, isomerization, and elimination through oxidative and β-oxidation-associated pathways, resulting in detectable metabolites in plasma, urine, and expired carbon dioxide (Ross et al., 2011). Elimination kinetics vary considerably, ranging from hours to days depending on tissue retention and redistribution dynamics (Paul et al., 2020). Excretion occurs predominantly as polar metabolites via urinary and respiratory pathways rather than as intact LYC.

Despite these pharmacokinetic constraints, LYC exhibits favorable toxicological characteristics and is generally considered safe even at relatively high intake levels (Tufail et al., 2024). Nevertheless, subtle alterations in biochemical parameters observed in some high-dose and nanoformulated experimental systems indicate that translational safety evaluation remains incomplete (Neves-Silva et al., 2025). Another pharmacologically relevant feature involves the ability of LYC to cross the blood-brain barrier, likely facilitated by its highly lipophilic nature (Paul et al., 2020). This property has stimulated growing interest in LYC as a neuroprotective agent targeting mitochondrial dysfunction in neurodegenerative diseases. However, quantitative analyses of brain accumulation, intracellular localization, and regional distribution remain scarce, representing an important limitation for translational neuroscience applications.

Beyond direct antioxidant activity, LYC exerts pleiotropic biological effects through modulation of signaling pathways involved in redox adaptation, inflammatory regulation, metabolism, and cell survival (Lian and Wang, 2008; Sharoni et al., 2012; Marzocco et al., 2021). Experimental studies indicate that LYC modulates pathways involving nuclear factor erythroid 2-related factor 2 (Nrf2), nuclear factor-κB (NF-κB), AMP-activated protein kinase/sirtuin 1/peroxisome proliferator-activated receptor gamma coactivator 1-α (AMPK/SIRT1/PGC-1α, respectively), phosphoinositide 3-kinase (PI3K)/Akt, Janus kinase/signal transducer and activator of transcription 3 (JAK/STAT3), and fibroblast growth factor 21 (FGF21)-associated signaling networks, thereby integrating mitochondrial regulation with broader cellular adaptation programs. Importantly, many of these signaling axes converge mechanistically at the mitochondrial level. Accordingly, LYC has been associated with preservation of mitochondrial membrane potential, attenuation of mitochondrial ROS generation, modulation of electron transport chain (ETC.) activity, stabilization of calcium homeostasis, and regulation of mitochondria-associated cell death pathways (Paul et al., 2020). Collectively, these observations position LYC at the intersection of redox adaptation, metabolic sensing, and mitochondrial resilience.

This mechanistic complexity becomes particularly relevant in the context of mitochondrial quality control, which encompasses mitochondrial biogenesis, fusion–fission dynamics, mitophagy, and mitochondrial redox homeostasis (Zhang et al., 2025). Although accumulating evidence indicates that LYC modulates these interconnected processes, much of the current literature still relies predominantly on indirect molecular markers rather than integrated structural, functional, and causality-driven analyses. Consequently, the mechanistic hierarchy underlying LYC action remains incompletely defined. At present, it remains uncertain whether LYC should be interpreted primarily as an organelle-level effector directly targeting mitochondrial components or as a broader adaptive metabolic regulator whose mitochondrial effects emerge secondarily from systemic redox and signaling reprogramming.

This uncertainty acquires additional translational significance because physiologically achievable concentrations of LYC are relatively low, whereas many *in vitro* studies employ micromolar concentrations substantially exceeding typical plasma levels (Liu et al., 2006). Even controlled human supplementation studies using oral doses around 10 mg produce transient plasma peaks accompanied by extensive metabolism and redistribution (Ross et al., 2011), suggesting that sustained intracellular accumulation may be difficult to achieve under physiological conditions. Consequently, improving LYC stability, absorption, and tissue targeting has become a major research priority. In this context, nanotechnology-based delivery systems (including liposomes, nanoemulsions, nanomicelles, and Pickering emulsions) have emerged as promising strategies capable of enhancing solubility, protecting against degradation, and improving tissue distribution (Li et al., 2025a; Neves-Silva et al., 2025). Notably, nanoformulated LYC frequently exhibits greater biological efficacy than conventional preparations, likely due to enhanced cellular uptake and intracellular delivery. Whether such systems can achieve selective mitochondrial targeting, however, remains largely unexplored.

Against this background, critical reassessment of how LYC influences distinct dimensions of mitochondrial physiology becomes particularly relevant. The present review therefore examines, in a mechanistically integrated manner, the effects induced by LYC on multiple aspects of mitochondrial biology across different experimental systems. Initially, the review discusses the effects of LYC on mitochondrial function and bioenergetic homeostasis. Subsequently, the effects of LYC on mitochondrial redox biology and mitochondria-associated cell death pathways are critically evaluated. The following sections address mitochondrial biogenesis, fusion-fission dynamics, and mitophagy, respectively, integrating these interconnected processes within a unified framework of mitochondrial quality control. Through this organization, the review seeks to determine whether LYC should be positioned as a direct regulator of mitochondrial physiology or as a broader systems-level modulator whose mitochondrial effects arise secondarily from integrated metabolic and redox adaptation. Importantly, the review also discusses the pharmacokinetic, translational, and mechanistic limitations that currently prevent definitive therapeutic positioning of LYC as a mitochondria-targeted intervention. Clarifying these relationships may ultimately redefine whether dietary bioactives such as LYC should be interpreted as direct organelle regulators or as systems-level modulators of cellular stress adaptation. The pro-apoptotic effects induced by LYC in tumor cells were not included because recent dedicated reviews have already addressed this topic in detail (Puah et al., 2021; Kapała et al., 2022; Maaz et al., 2025; Yin et al., 2025).

## 2 The effects on mitochondrial physiology

Despite increasing evidence linking LYC to mitochondrial protection, the mechanistic interpretation of these effects remains complex. In many experimental systems, mitochondrial improvements occur concomitantly with modulation of inflammatory signaling, redox homeostasis, metabolic adaptation, and inter-organelle communication, making it difficult to determine whether mitochondria represent primary molecular targets of LYC or secondary beneficiaries of broader cellular reprogramming. This complexity is further amplified by the dynamic nature of mitochondrial biology, in which bioenergetics, redox regulation, mitochondrial biogenesis, fusion-fission dynamics, and mitophagy operate as highly interconnected processes rather than isolated events.

Importantly, the mitochondrial effects induced by LYC appear to be strongly context dependent, varying according to tissue type, pathological condition, metabolic state, and experimental exposure paradigm. In some models, LYC predominantly preserves mitochondrial respiration and membrane potential, whereas in others it modulates mitochondrial turnover, stress signaling, or organelle network organization. Against this background, the following sections critically examine the effects of LYC on distinct dimensions of mitochondrial physiology, integrating these processes within a unified framework of mitochondrial adaptation and quality control.

### 2.1 Effects of LYC on mitochondrial function

**FIGURE 1:**
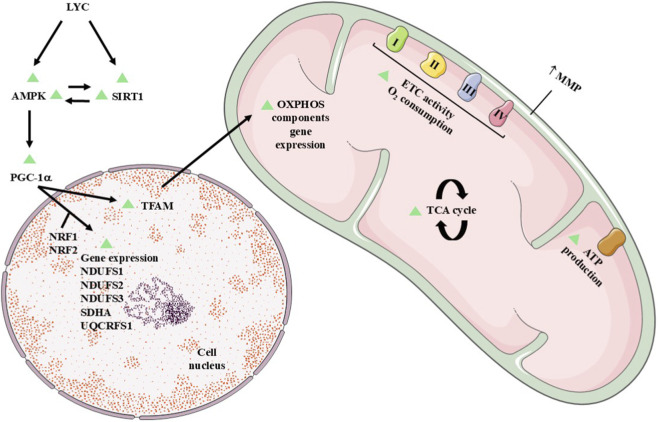
A summary of the effects promoted by LYC on mitochondrial function. LYC activates the AMPK/SIRT1/PGC-1α signaling axis, promoting NRF1/NRF2-dependent expression of TFAM and OXPHOS-related genes. Increased expression of OXPHOS components may enhance the activity of respiratory Complexes I - V, improving MMP, oxygen consumption, and ATP production. In parallel, LYC appears to stimulate TCA cycle enzymes, potentially increasing substrate availability for mitochondrial respiration. Collectively, these effects support improved mitochondrial bioenergetic performance across different experimental models. Evidence further suggests that these bioenergetic adaptations may be associated with induction of mitochondrial biogenesis, although mechanistic validation remains limited. Please, read the text and analyze the tables for more detailed information. This figure exhibits images created by Servier Medical Art, which are licensed under a Creative Commons Attribution 4.0 Unported License (https://creativecommons.org/licenses/by/4.0/).

LYC has been consistently associated with improvements in mitochondrial physiology across multiple experimental systems; however, a critical examination of the current literature indicates that much of the available evidence still relies on associative biochemical endpoints rather than mechanistically resolved mitochondrial analyses (Figure 1; Table 1). Across tissues, LYC appears to converge on three interconnected regulatory dimensions: redox homeostasis, metabolic adaptation, and organelle communication. Nevertheless, the relative contribution of direct organelle-level modulation versus broader cellular and systemic adaptations remains insufficiently defined. This distinction is particularly relevant because many studies report restoration of mitochondrial function concomitantly with modulation of inflammatory, antioxidant, and metabolic pathways, making it difficult to establish mechanistic hierarchy.

**TABLE 1:** The effects of LYC on mitochondrial function.

Biological target	Experimental model	Major findings	References
Rat H9c2 cardiomyocytes and C57BL/6J mice heart and plasma	*In vitro*: DT at 10 µM for 72 h in the presence or not of LYC at 5 µM *In vivo*: mice were administrated with DT at 420 μg/kg.day^-1^ body weight in the presence or absence of LYC at 5 or 10 mg/kg.day^-1^ through gavage for 35 days	*In vitro*: Restored cell viability; reduced ROS production; diminished CK and LDH release; attenuated MDA levels; reduced the mRNA levels of UCP2 and UCP3 Increased the levels of acetyl-CoA and ATP; decreased the NADH/NAD^+^ ratio; stimulated Ca^2+^/Mg^2+^-ATPase activity; increased MMP; restored the expression of genes associated with mitochondrial complexes (*e.g.*, ND2, ND3, ND5, SDHA, UQCRFS1, COX2, and ATPef08); decreased the levels of FFA and the expression of CD36; downregulated the expression of ACC1; upregulated PPARα and ACOX1 expression; attenuated the expression of CPT-I and LCAD; increased mtDNA copy number; did not alter TFAM expression; restored the expression of AMPK, PGC-1α, and SIRT1; increased the activity of complexes I and IV; failed to restore the activity of complexes III and V *In vivo*: Increased the cardiac levels of acetyl-CoA and ATP; enhanced NADH/NAD^+^ ratio; restored Ca^2+^/Mg^2+^-ATPase and na^+^/K^+^-ATPase activity; restored mitochondrial ultrastructure Plasma FFA levels did not change; decreased heart FFA content; reduced CK and LDH activity in plasma; suppressed cardiac hypertrophy Diminished the levels of DT and AOPP in heart tissue; decreased ROS levels in both plasma and heart; restored heart SOD activity; augmented GSH/GSSG ratio; upregulated heart CAT, GPX, and Mn-SOD activity; stimulated the expression of Nrf2, HO-1, and NQO1	Wang et al. (2022a)
Rat liver	LYC at 10 mg/kg body weight (i.p. injection) for 6 days prior to the induction of FHF stimulated by D-GalN/ LPS at 300 mg and 30 μg/kg body weight, respectively, 18 h before rat sacrifice (i.p. injected)	Restored the activity of IDH, α-KGDH, SDH, and MDH; enhanced the activity of the complexes I and IV; increased the levels of ATP Decreased mitochondrial lipid peroxidation; reduced mitochondrial H_2_O_2_ production; conserved the levels of SOD, GPX, GST, and GSH Docking studies suggest that LYC inhibits lipoxygenase enzymes	Sheriff et al. (2017)
Murine 3T3-L1 preadipocytes (differentiated into adipocytes), rat primary adipocyte, and male ICR mice adipose tissue	*In vitro*: 3T3-L1 cells: LYC at 1, 2, or 4 µM in the presence or not of GW9662 (at 20 µM – inhibitor of PPARγ) for 6–8 days Primary adipocyte isolated from the rat eWAT: LYC at 1, 2, or 4 µM in the presence or not of GW9662 (at 10 µM) during differentiation and maturation *In vivo*: obese ICR mice received LYC at 15 mg/kg for 10 weeks (orally administrated)	*In vitro*: 3T3-L1 cells: Stimulated glucose consumption and glycerol release by a PPARγ-dependent manner; upregulated UCP1, PRDM16, and PGC-1α protein levels; upregulated PPARγ Primary adipocytes: Stimulated glucose consumption and glycerol release by a PPARγ-dependent manner; enhanced basal mitochondrial respiration, ATP-linked respiration, maximal mitochondrial respiratory capacity, spare (nonmitochondrial) respiratory capacity, and uncoupling capacity (PPARγ-dependent mechanism); upregulated UCP1, PRDM16, and PGC-1α (mRNA and protein levels); upregulated PPARγ *In vivo*: decreased body weight gain; reduced body fat mass; diminished adipocyte size; decreased serum LDL, total cholesterol, triglycerides, ALT and AST levels; restored serum insulin levels and glycemia; reduced fasting blood glucose levels; upregulated UCP1, PRDM16, PGC-1α, and PPARγ (mRNA and protein levels) in eWAT (similar effects were seen in iBAT)	Zhu et al. (2020)
Mice hippocampus and cerebral cortex	LYC at 2.5–10 mg/kg.day^-1^ (i.p. injected) during the exposure to PTZ at 40 mg/kg (i.p. Injected at alternate days) for 29 days	Improved mice behavior; stimulated complexes I, II, and IV activity; decreased lipid peroxidation and nitrite levels; enhanced GSH content and SOD and CAT activity	Bhardwaj and Kumar (2016)
Wistar rat brain	LYC at 2.5 and 5 mg/kg (oral administration) after the administration of Aβ_1-42_ at 3 nmol/3 µL (intracerebroventricular administration)	Improved cognitive function; restored complexes I, II, III, and IV activity; decreased MDA and nitrite levels; enhanced the activity of SOD and CAT and augmented the concentration of GSH; stimulated acetylcholinesterase activity; reduced the levels of TNF-α and IL-6	Prakash and Kumar (2014)
CD-1 mice hippocampus	LYC at 0.03% w/w (mixed with standard chow) in the presence of D-gal at 150 mg/kg.day^-1^ (i.p. injected) for 9 weeks	Increased phospho-AMPK, SIRT1, NDUFS1, and NDUFS3 protein levels Ameliorated working memory; decreased the number of nuclei pyknosis and necrosis; improved length and width of postsynaptic density; reduced β-galactosidase activity; upregulated SNAP-25, PSD-95, and BDNF protein levels; downregulated P19, P21, and P53 protein levels (senescence-associated proteins) Increased SOD and CAT activity and GSH levels; downregulated phospho-JNK, phospho-ERK, phospho-p38, IL-1β, and TNF-α protein levels	Wang et al. (2023a)
HT-22 and HepG2 cells and CD-1 mice hippocampus and cerebral cortex	*In vitro*: HepG2-HT-22 co-culture: HepG2 cells were treated with LYC and D-gal for 8 h Recombinant human FGF21 at 100 nM was administrated for 8 h *In vivo*: LYC at 0.03% w/w (mixed with standard chow were administrated to aged mice (15-month-old) for 3 months AAV-shFGF21 was administrated at 5 ×10^9^ plaque-forming units viruses per mouse (tail vein injection)	*In vitro*: HT22-HepG2 cells co-culture exposed to D-gal: Increased FGF21 protein levels; restored ATP levels; increased axon length; decreased mitochondrial production of reactive species; suppressed loss of MMP HT22 cells: Purified FGF21 induced similar effects on mitochondrial function when compared to LYC *In vivo*: Hippocampus: LYC restored BDNF positive area in CA1, CA3, and DG; upregulated PSD-95 protein levels; attenuated mitochondrial swelling and vacuolation Cerebral cortex: Restored BDNF positive area and the protein levels of BDNF and NGF; increased the length and width of post synaptic density; upregulated PSD-95 and SNAP-25 protein levels; enhanced the levels of syntaxin bound to SNAP-25; increased the protein levels of VAMP; augmented the levels of VAMP bound to SNAP-25 Restored the protein levels of NDUFS1, NDUFS2, NDUFS3, and ATP synthase; increased the levels of ATP Downregulated IL-1β, COX-2, IL-6, and TNF-α Upregulated Nrf2 and HO-1 protein levels; improved SOD and CAT enzyme activity and augmented reduced GSH levels; decreased H_2_O_2_ levels; enhanced FGF21 protein levels Plasma: Increased SOD and CAT enzyme activity; failed to modulate plasma GSH; increased levels of FGF21 Liver: Upregulated PPARα and FGF21	Wang et al. (2024b)
Rat cerebellar granule neurons	LYC at 10 µM for 2 h before the challenge with MeHg at 500 nM for further 12 h	Prevented loss of cell viability and LDH release Decreased total and mitochondrial ROS production; attenuated loss of MMP and mPTP opening; prevented complexes III and IV activity decline and ATP levels reduction; attenuated COX1 and ND6 expression decrease	Qu et al. (2013)
SPF mice cerebellum	LYC at 5 mg/kg (body weight) to mice treated with DEHP at 500 mg/kg (body weight) (both intragastrically administrated) for 28 days	Restored the number of purkinje cells; reduced cerebellar Ca^2+^ content; stimulated the expression of VMP1, IP3R1, and SERCA2 Downregulated MFN2; restored the co-localization of IP3R1 and calbindin (similar effects were seen regarding SERCA2 and calbindin); suppressed UPR^ER^; decreased the levels of CLPP, LONP1, and ATF5 (attenuated UPR^mt^); restored mitochondrial cristae and membranes structure; attenuated mitochondrial vacuolization and volume density	Cui et al. (2024)
Intestinal porcine enterocytes IPEC-J2 cell line (intestinal porcine enterocytes obtained from the jejunum of neonatal piglet)	LYC at 5–15 µM for 4 or 6 h before the administration of DON at 1 μg/mL for further 48 h (berberine at 20 µM was utilized to downregulate the expression of genes associated with OXPHOS)	Restored cell viability; reduced the number of apoptotic cells; attenuated ROS levels; restored SOD and CAT activity; decreased LDH leakage; restored MMP, mtDNA copy number, and ATP levels Berberine abrogated the antioxidant action and cytoprotective effects promoted by LYC	Wang et al. (2024a)

Cardiac models provide some of the most detailed evidence linking LYC to bioenergetic remodeling. In doxorubicin-associated cardiotoxicity, LYC attenuated oxidative injury while partially restoring mitochondrial respiratory activity (Wang et al., 2022a). The preferential recovery of Complexes I and IV, together with persistent dysfunction of Complexes III and V, suggests selective stabilization of electron entry and terminal electron transfer rather than complete restoration of, ETC integrity. This profile is more compatible with attenuation of upstream electron leak and ROS generation than with full normalization of oxidative phosphorylation (OXPHOS) efficiency (Murphy, 2009; Brand, 2016; Robb et al., 2018). Likewise, activation of the AMPK/SIRT1/PGC-1α axis was inferred primarily from transcriptional modulation, without direct assessment of post-translational activation states (e.g., SIRT1-mediated deacetylation of PGC-1α) or mitochondrial biogenesis flux. Consequently, whether LYC induced genuine expansion of mitochondrial mass or instead enhanced the performance of pre-existing organelles cannot yet be mechanistically prioritized. Importantly, the concomitant modulation of lipid metabolism [including suppression of fatty acid translocase (CD36) and acetyl-CoA carboxylase 1 (ACC1) together with induction of peroxisome proliferator-activated receptor-α (PPARα) and acyl-coenzyme A oxidase 1 (ACOX1)] further suggests that cardiac mitochondrial improvements may emerge from coordinated remodeling of substrate utilization rather than isolated effects on respiratory complexes.

A related interpretative complexity emerges in intestinal epithelial systems. Under deoxynivalenol (DON)-induced stress, LYC preserved mitochondrial membrane potential (MMP) and ATP production while reducing oxidative damage (Wang et al., 2024a). Although suppression of OXPHOS-associated genes by berberine suggested mitochondrial involvement, this pharmacological strategy did not resolve whether LYC altered substrate oxidation, electron transfer kinetics, or proton leak dynamics. Given the marked sensitivity of epithelial tissues to cytosolic redox imbalance, an alternative explanation is that restoration of antioxidant buffering secondarily preserved mitochondrial performance (Guerbette et al., 2022). Distinguishing between compartment-specific antioxidant actions and direct bioenergetic modulation will require integrated analyses of mitochondrial respiration, redox compartmentalization, and metabolite flux.

In hepatic systems, LYC reduced mitochondrial hydrogen peroxide (H_2_O_2_) generation and enhanced the activity of multiple dehydrogenases linked to TCA cycle metabolism (Sheriff et al., 2017), findings compatible with improved coordination between substrate oxidation and respiratory activity. Nevertheless, the absence of direct interrogation of Nrf2 transcriptional activity, NF-κB signaling dynamics, or ETC-associated ROS production restricts mechanistic attribution. Similarly, docking analyses suggesting inhibition of lipoxygenase enzymes remain speculative in the absence of biochemical validation. These limitations complicate interpretation of whether the observed mitochondrial benefits resulted from direct modulation of mitochondrial enzymes or from broader attenuation of inflammatory and lipid peroxidation pathways. Importantly, because the liver exerts systemic metabolic control, hepatic adaptations induced by LYC may influence mitochondrial function in distal tissues through endocrine or metabolite-dependent signaling, a concept later reinforced by studies involving FGF21 (Prida et al., 2022).

Adipose tissue models position LYC as a potential regulator of mitochondrial oxidative capacity through peroxisome proliferator-activated receptor-γ (PPARγ)-dependent metabolic reprogramming (Zhu et al., 2020). Induction of uncoupling protein 1 (UCP1), PR domain zinc finger protein 16 (PRDM16), and PGC-1α is consistent with acquisition of a thermogenic phenotype enriched in mitochondria. However, the mechanistic hierarchy underlying these effects remains incompletely resolved. Although GW9662-supported inhibition of PPARγ signaling demonstrated pathway involvement, it did not establish pathway sufficiency or exclude parallel regulatory mechanisms. Moreover, putative mitochondrial remodeling was inferred primarily from transcriptional markers rather than direct quantification of organelle expansion, mitochondrial DNA (mtDNA) replication, or network remodeling. The increase in uncoupling capacity introduces an additional level of complexity, as enhanced proton conductance may reflect adaptive thermogenesis but may also indicate controlled bioenergetic inefficiency (Papa et al., 2012). Thus, whether LYC improves mitochondrial quality or shifts cellular metabolism toward regulated energetic dissipation remains uncertain.

Neurobiological systems reveal a broader integration between mitochondrial regulation, synaptic maintenance, and systemic signaling. Across seizure (Bhardwaj and Kumar, 2016), aging (Prakash and Kumar, 2014; Wang et al., 2023a; Wang et al., 2024b), and neurotoxicity (Qu et al., 2013) models, LYC restored, ETC activity, ATP production, and antioxidant defenses while attenuating inflammatory mediators and neuronal degeneration. However, most observations derive from static biochemical measurements, limiting interpretation of dynamic mitochondrial processes such as respiratory coupling efficiency, mitochondrial turnover, or network plasticity. In aging-associated models, increased expression of ETC-associated proteins, including NADH-ubiquinone oxidoreductase core subunit (NDUFS) subunits (Wang et al., 2023a), was interpreted as evidence of improved mitochondrial function; yet without direct quantification of oxygen consumption or ATP synthesis efficiency, these changes may also represent compensatory responses to persistent metabolic stress. Moreover, the relationship between suppression of neuroinflammation and restoration of mitochondrial performance remains insufficiently delineated. This distinction is particularly relevant because inflammatory signaling pathways such as c-Jun N-terminal kinase (JNK), extracellular signal-regulated kinase (ERK), and p38 directly influence mitochondrial physiology and neuronal survival (Peggion et al., 2024; Yu et al., 2025).

A more mechanistically integrated framework emerged from studies identifying a liver–brain endocrine axis mediated by FGF21 (Wang et al., 2024b). The use of AAV-shFGF21 provided important causal evidence indicating that LYC-induced neuroprotection depends, at least partially, on hepatic signaling. Nevertheless, the downstream mitochondrial targets of FGF21 within neuronal tissues remain poorly characterized. It is unclear whether FGF21 preferentially modulates mitochondrial biogenesis, substrate utilization, antioxidant defenses, or synaptic bioenergetics. Likewise, potential interactions between FGF21 signaling and canonical mitochondrial regulators such as AMPK, SIRT1, or Nrf2 were not explored experimentally, leaving a mechanistic discontinuity between endocrine regulation and organelle adaptation. An additional unresolved question concerns the relationship between activation of the Nrf2/heme oxygenase-1 (HO-1) axis and the mitochondrial, redox, and immune alterations observed in LYC-treated neural tissues. Given the central role of Nrf2 and HO-1 in mitochondrial adaptation, antioxidant defense, and immune regulation (Holmström et al., 2016; Carr et al., 2020; Saso et al., 2025), determining whether these pathways are necessary for LYC-mediated neuroprotection represents an important future direction.

At the level of organelle communication, cerebellar studies suggest that LYC modulates mitochondria-endoplasmic reticulum (ER) crosstalk, particularly through regulation of Ca^2+^ trafficking via inositol 1,4,5-trisphosphate receptor type 1 (IP3R1) and sarcoplasmic/endoplasmic reticulum calcium ATPase 2 (SERCA2) (Cui et al., 2024). The attenuation of both reticular and mitochondrial unfolded protein responses (UPR^ER^ and UPR^mt^, respectively), together with preservation of mitochondrial ultrastructure, supports the interpretation that LYC reduces proteostatic stress within interconnected organelle networks. However, the proposed remodeling of mitochondrial dynamics through mitofusin 2 (MFN2) downregulation remains speculative in the absence of direct assessment of fusion/fission kinetics or mitochondrial turnover (Sun et al., 2015). Because Ca^2+^ transfer at mitochondria-associated membranes (MAMs) critically regulates metabolism, apoptosis, and stress adaptation (Bernardi et al., 2023), this interface may represent one of the most relevant yet underexplored mechanistic dimensions of LYC action.

Similarly, in methylmercury-induced neurotoxicity, LYC prevented mitochondrial permeability transition pore (mPTP) opening while preserving ATP levels and ETC activity (Qu et al., 2013). Yet the absence of analyses involving cyclophilin D regulation, mitochondrial Ca^2+^ dynamics, or redox-sensitive thiol modifications prevents hierarchical interpretation of how LYC stabilized mitochondrial permeability under oxidative stress conditions. Consequently, it remains uncertain whether the primary target involved ETC-derived oxidative burden, thiol oxidation, membrane lipid preservation, or regulation of permeability transition machinery itself.

Despite substantial consistency across models, several methodological limitations continue to restrict mechanistic interpretation. Many studies rely predominantly on mRNA expression profiles or isolated enzyme-activity assays without comprehensive protein-level confirmation or post-translational analyses. In addition, dynamically resolved bioenergetic parameters (including respiratory control ratios, proton leak kinetics, coupling efficiency, and compartment-specific mitochondrial ROS generation) remain insufficiently explored. Similarly, direct assessment of mitochondrial turnover, respiratory supercomplex organization, and real-time fusion/fission dynamics is still scarce despite recurrent alterations in MFN2, ETC subunits, and stress-response pathways. Another critical limitation involves the absence of pharmacokinetic and subcellular distribution analyses, which prevents determination of whether LYC accumulates within mitochondrial membranes at concentrations compatible with direct interaction with, ETC complexes, membrane lipids, or signaling proteins. Collectively, these limitations hinder establishment of causal continuity between LYC exposure, mitochondrial remodeling, and physiological outcomes. Advancing the field will therefore require transition from descriptive mitochondrial biochemistry toward integrated mitochondrial physiology combining multi-omics, high-resolution bioenergetics, real-time imaging, and rigorous loss- and gain-of-function approaches.

Taken together, current evidence indicates that LYC exerts broad regulatory effects on mitochondrial physiology through coordinated modulation of redox balance, metabolic signaling, bioenergetic adaptation, and organelle communication. Its capacity to preserve ATP synthesis, stabilize calcium homeostasis, attenuate oxidative stress, and maintain mitochondrial ultrastructure across heart, liver, intestine, adipose tissue, hippocampus, cortex, and cerebellum highlights substantial translational potential. Nevertheless, the predominance of associative evidence continues to complicate definitive mechanistic classification. At present, LYC cannot be unequivocally categorized as a direct mitochondrial effector, a transcriptional regulator, a systemic metabolic modulator, or a mitochondria-targeted therapeutic compound, because available data support features of all these mechanisms without resolving their relative hierarchy. Future investigations integrating spatially resolved mitochondrial analyses, pharmacokinetic characterization, and causal pathway interrogation will be essential to determine whether LYC functions primarily through direct organelle modulation or through systems-level metabolic adaptation. Only after these mechanistic relationships are clarified will it become possible to define the true therapeutic positioning of LYC as a modulator of mitochondrial dysfunction across multiple pathological contexts.

### 2.2 Effects of LYC on mitochondria-related redox biology and cell death

**FIGURE 2:**
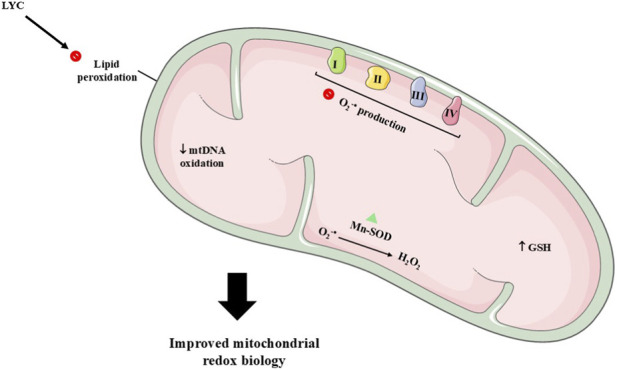
A summary of the effects promoted by LYC on the mitochondrial redox biology. Through mechanisms not yet fully understood, LYC is able to decrease the production of O_2_^−•^ and H_2_O_2_ in mitochondria of different cell types. Apparently, the decrease in O_2_^−•^ production may be associated with the beneficial effects promoted by LYC on the activity of, ETC complexes. Furthermore, LYC is able to stimulate the activity of the Mn-SOD enzyme, which converts O_2_^−•^ into H_2_O_2_, decreasing its mitochondrial levels. Together, these effects may favor a decrease in mtDNA oxidation through the reduced production of hydroxyl radical (^•^OH), a product of the reaction between O_2_^−•^ and H_2_O_2_ (among other origins). LYC has also been shown to cause an increase in GSH levels and protein and total thiols. LYC can stimulate the Nrf2 transcription factor (not shown in the figure), but there is no clear evidence of the relationship of this modulation to mitochondrial redox biology. Furthermore, the mitochondrial redox action promoted by LYC appears to be related to the inhibition of cell death processes such as apoptosis (intrinsic pathway), cuproptosis, and pyroptosis, although this has not yet been fully clarified. Please, read the text and analyze the tables for more detailed information. This figure exhibits an image created by Servier Medical Art, which is licensed under a Creative Commons Attribution 4.0 Unported License (https://creativecommons.org/licenses/by/4.0/).

LYC has been extensively characterized as a modulator of mitochondria-centered redox homeostasis and cell death (Figure 2; Table 2). However, the prevailing narrative remains largely inferential, with most studies relying on convergent downstream phenotypes rather than direct interrogation of mitochondrial mechanisms. Across experimental systems, LYC consistently suppresses oxidative stress, stabilizes MMP, and attenuates cell death execution, yet these endpoints do not distinguish primary mitochondrial targeting from upstream modulation of redox-sensitive signaling networks. Mechanistically, current evidence supports a model in which LYC operates at the interface of mitochondrial ROS production, redox-responsive transcriptional programs (e.g., Nrf2 and NF-κB), inflammatory signaling, and cell death-regulatory checkpoints, although the causal hierarchy among these processes remains incompletely defined. This ambiguity is further compounded by the absence of quantitative data regarding intramitochondrial LYC accumulation and molecular binding targets, precluding definitive assignment of mechanistic specificity.

**TABLE 2:** The effects of LYC on mitochondria-related redox biology and cell death.

Biological target	Experimental model	Major findings	References
Human neuroblastoma SH-SY5Y cells	LYC at 2 µM for 2 h before the challenge with MPTP at 500 µM for additional 24 h	Decreased mitochondrial production of O_2_^−•^; diminished cellular ROS production and MDA levels Prevented loss of cell viability; decreased number of apoptotic cells; prevented MMP collapse; suppressed mPTP opening; restored ATP levels; stimulated COX1 and ND6 gene expression	Yi et al. (2013)
Human neuroblastoma SH-SY5Y cells	LYC at 2 or 4 µM for 2 h prior to the exposure to H_2_O_2_ at 400 µM for further 24 h	Reduced ROS production; stimulated SOD and CAT activity Prevented loss of cell viability and cytotoxicity; decreased the number of apoptotic cells; upregulated Bcl-2; downregulated bax; attenuated mPTP opening; blocked cytochrome c release; prevented caspase-3 activation; suppressed nuclear translocation of AIF	Feng et al. (2016)
Human neuroblastoma SH-SY5Y cell line	LYC at 0.2 or 0.5 µM for 1 h prior to the administration of Aβ at 20 µM for additional 24 h	Decreased total and mitochondrial ROS production Prevented MMP decline and OCR decrease; prevented cell number decline; increased Bcl-2 and decreased bax protein levels; reduced p53 protein levels; diminished caspase-3 activation; reduced phosphorylation of IκB; inhibited NF-κB binding activity; reduced nucling immunocontent	Hwang et al. (2017)
Primary rat hippocampal neurons	LYC at 1 µM for 2 h prior to the exposure to TMT at 5 µM for further 24 h	Decreased cellular ROS production; diminished mitochondrial O_2_^−•^ production Prevented loss of cell viability; reduced number of apoptotic cells; attenuated mPTP opening; prevented MMP decline; reduced cytochrome c release; prevented caspase-3 activation	Qu et al. (2011)
Primary rat cortical neurons	LYC at 2 µM for 4 h prior to the exposure to amyloid β_1-42_ at 10 µM for up to 24 h	Decreased cellular and mitochondrial ROS production; decreased mitochondrial DNA oxidation Prevented complexes I, II, III, and IV activity decline; restored ATP content; enhanced COX1 and ND6 gene expression; augmented TFAM protein levels Attenuated mPTP opening; reduced cytochrome c release	Qu et al. (2016)
Primary mouse cerebrocortical neurons	LYC at 4 µM for 4 h prior to the exposure to *t*-BHP at 10 µM for additional 24 h	Attenuated GSH depletion; increased GSH/GSSG ratio; reduced ROS production Prevented loss of cell viability; conserved MMP; attenuated caspase-3 activation and number of apoptotic cells; decreased bax protein levels and cytochrome c release; upregulated Bcl-2 Increased SYP and PSD-95 protein levels; activated PI3K/Akt signaling pathway	Huang et al. (2019)
Primary rat hippocampal neurons	LYC at 5 µM for 4 h before the administration of lead at 50 µM for further 24 h	Decreased total and mitochondrial ROS production Attenuated mPTP opening; restored complexes I, II, III, and IV activity and MMP levels; prevented ATP levels decline Increased Bcl-2 and decreased bax protein levels; attenuated cytochrome c release; reduced caspase-3 activation; prevented loss of cell viability; reduced the number of apoptotic cells	Qu et al. (2020)
Rat spinal cord	LYC at 5–20 mg/kg.day^-1^ (i.p. injected) prior to SCI [Duration of LYC treatment was not described]	Attenuated lipid peroxidation; stimulated SOD and GPX activity Increased cytochrome b and Tfam gene expression Prevented loss of MMP; increased Bcl-2 protein levels; attenuated cytochrome c release; decreased bax, caspase-3, and caspase-9 immunocontents; reduced the number of apoptotic cells Improved locomotor function	Hu et al. (2017)
Wistar rat striatum	LYC at 10 mg/kg.day^-1^ (oral administration) during the exposure to 3-NP at 25 mg/kg (i.p. injected) for four consecutive days	Decreased mitochondrial ROS production and nitrite levels; diminished mitochondrial lipid peroxidation; restored Mn-SOD activity; enhanced the mitochondrial levels of total thiols, low molecular weight thiols, and protein thiols Restored complexes II, IV, and V activity; attenuated mitochondrial swelling and cytochrome c release; downregulated caspase-3 and p53 Improved locomotory performance	Sandhir et al. (2010)
Wistar rat hippocampus and cerebral cortex	LYC at 2.5 or 5 mg/kg (oral administration) for 21 days after the administration of colchicine at 15 µg/5 µL (intracerebroventricular administration)	Decreased MDA and nitrite levels; increased SOD and CAT activity and GSH levels Restored complexes I, II, and IV activity Reduced IL-6 and TNF-α levels; decreased caspase-3 activity Decreased acetylcholinesterase activity; improved cognitive function	Prakash and Kumar (2013)
Primary C57BL/6 mice cardiomyocyte	LYC at 5 µM 4 h before exposure to hypoxia followed by reoxygenation (for up to 16 h each) injury	Decreased ROS production and MDA levels Attenuated mPTP opening; prevented loss of MMP and ATP levels decline; diminished cytochrome c release and caspase-3 activity; prevented loss of cell viability and apoptosis rate	Yue et al. (2012)
Rat cardiomyocyte H9c2 cells and rat heart	*In vitro*: LYC at 10 µM for 12 h before the induction of hypoxia (for 12 h) and reoxygenation (for 1 h) *In vivo*: LYC at 40 mg/kg.day^-1^ for 5 days prior to the heart isolation and induction of ischemia (for 30 min) and reperfusion (for 120 min) injury	*In vitro* Downregulated bax and upregulated Bcl-2; prevented MMP decline; decreased cytochrome c release, APAF1 levels, and activation of caspase-9 and caspase-3; conserved cell viability; decreased apoptosis rate *In vivo*	Li et al. (2019)
​	​	Reduced mPTP opening; decreased apoptosis rate; decreased cytochrome c release, APAF1 levels, and activation of caspase-9 and caspase-3; downregulated bax and upregulated Bcl-2; reduced infarct size Atractyloside (inducer of mPTP opening) suppressed the mitochondria-related anti-apoptotic actions promoted by LYC	​
Chicken heart	LYC at 5 mg/kg during the exposure to ATZ at 100 mg/kg (both added to the diet) for 4 months	Lowered copper content; augmented SOD and CAT enzyme activity; reduced H_2_O_2_ and MDA levels; increased NADPH amounts Attenuated mitochondrial cristae fragmentation and vacuolization; restored mitochondrial number, MMP and the levels of pyruvate, α-ketoglutarate, and NADH Downregulated CTR1, FDX1, LIAS, DLAT, COX17, and ATP7A gene expression (mRNA levels); decreased lipoylated-DLAT, total DLAT, LIAS, CTR1, and COX17 protein levels; attenuated β-galactosidase enzyme activity; downregulated CDKN1A, CDKN2A, and γ-H2AX protein content; upregulated lamin B1 protein levels Decreased MMP3, IL-8, IL-1β, and IL-6 gene expression (mRNA levels) Improved cardiac function	Li et al. (2025b)
Rat cardiac microvascular endothelial cells (CMECs) and sprague-dawley rat heart	*In vitro* LYC at 5 µM after induction of hypoxia (for 12 h) and reoxygenation (for 24 h) *In vivo* LYC at 25–100 mg/kg.day^-1^ (through gavage) for 14 days after induction of hypoxia (for 45 min) and reoxygenation (for 6 h)	*In vitro* Improved cell viability; increased NO levels and CD31 protein content; enhanced Ang-2 and PDGFR-β gene expression (mRNA levels) Decreased cytosolic levels of mtDNA; increased ATP content Diminished IL-1β, IL-6, and IL-18 levels; downregulated cleaved-caspase-1 and GSDMD-terminal protein levels Downregulated E2F8 protein levels; silencing of FABP3 induced similar effects when compared to LYC; overexpression of E2F8 or FABP3 suppressed the benefits induced by LYC; overexpression of E2F8 increased FABP3 mRNA and protein levels; silencing of FABP3 suppressed the effects caused by E2F8 overexpression; downregulated YTHDF1 protein levels; silencing of YTHDF1 induced similar effects when compared to LYC; inhibited the YTHDF1/E2F8/FAPB3 axis and the cGAS-STING pathway	Sha et al. (2026)
​	​	*In vivo* Decreased cytosolic mtDNA levels; increased ATP content; reduced ROS production Attenuated IL-1β, IL-6, and IL-18 levels; diminished cleaved-caspase-1 and GSDMD-terminal protein levels Enhanced CD31 and phosphorylated-eNOS protein levels; increased Ang-2 and PDGFR-β gene expression (mRNA levels); stimulated NO production Downregulated FABP3 gene expression (mRNA levels) Improved cardiac function	​
Human hepatoma HepG2cell lines: 2E1 cells and neo cells	LYC at 10 µM for 2 h prior to the exposure to ethanol at 100 mM for up to 5 days	2E1 cells – transfected with human CYP2E1 Increased the mitochondrial levels of GSH; attenuated ROS production; the authors found a negative correlation between mitochondrial GSH and apoptosis rate; prevented loss of cell viability; reduced the number of apoptotic cells Neo cells – transfected with the pCI^-neo^ vector, an empty vector Failed to prevent loss of cell viability and apoptosis; did not alter cellular or mitochondrial GSH content	Xu et al. (2003)
LMH cells and chicken liver	*In vitro* LYC at 2.5 µM in the presence of ATZ at 200 µM for 12 h *In vivo* LYC at 5 mg/kg during the exposure to ATZ at 100 mg/kg (both added to the diet) for 4 months	*In vitro* Improved cell viability; restored the levels of pyruvate and α-ketoglutarate; downregulated CTR1, DLAT, FDX1, and ATOX1 gene expression (mRNA levels) and protein content; reduced lipoylated-DLAT content *In vivo* Attenuated ultrastructural changes; attenuated mitochondrial cristae fragmentation and vacuolization; restored the levels of pyruvate and α-ketoglutarate; Lowered copper content and MDA and H_2_O_2_ levels; stimulated SOD and CAT enzyme activity; increased GSH levels; downregulated CTR1, FDX1, LIAS, DLAT, COX17, and ATP7B gene expression (mRNA levels) and protein levels; reduced lipoylated-DLAT content; downregulated ATOX1 gene expression (mRNA levels) and protein content	Li et al. (2026)
Rat pancreatic acinar AR42J cells	LYC at 0.1 or 0.2 µM for 2 h prior to the exposure to ethanol (150 µM)/palmitoleic acid (50 µM) for up to 24 h	Decreased total and mitochondrial ROS production; reduced NADPH oxidase activity; prevented loss of MMP and ATP levels decline Downregulated NF-κB; attenuated trypsin and chymotrypsin activity; reduced IL-6 gene expression and protein levels; similar effects were induced by NAC or ML171 (NADPH oxidase inhibitor) Alleviated molecular effects seen in pancreatitis	Lee et al. (2021)
Kunming mice spleen	LYC at 5 mg/kg.day^-1^ during exposure to AFB_1_ at 0.75 mg/kg.day^-1^ (both orally administrated) for 30 days	Attenuated spleen injury; restored lymphocytes ratio (increased the number of CD3^+^, CD4^+^, and CD8^+^ T lymphocytes and restored IL-2, IFN-γ, and TNF-α levels, alleviating immunosuppression caused by AFB_1_) Reduced ROS production; decreased H_2_O_2_ levels; reduced MDA content; stimulated SOD and CAT enzyme activity Elevated Bcl-2 and decreased bax gene expression and protein levels; restored MMP; decreased cytochrome c release; attenuated caspase-3 gene expression and activation; reduced caspase-9 gene expression; decreased apoptosis rate	Xu et al. (2019)
Rat testicles and sperm	LYC at 4 mg/kg.day^-1^ (oral administration) 24 h before exposure to LPS at 0.1 mg/kg.day^-1^ (i.p. injection) for 7 days	Reduced H_2_O_2_ production; decreased MDA levels; increased the activity of mitochondrial SOD, CAT, GPX, GR, and ADH; enhanced mitochondrial GSH and vitamin C levels Increased the activity of SDH, MDH, and IDH Restored sperm number and motility	Aly et al. (2012)
Kunming mice testicles	LYC at 5 mg/kg.day^-1^ (oral administration) during the exposure to AFB_1_ at 0.75 mg/kg.day^-1^ (oral administration) for 30 days	Decreased H_2_O_2_ production and lipid peroxidation; promoted nuclear translocation of Nrf2; increased HO-1, NQO1, CAT, and SOD1 gene expression; stimulated SOD and CAT activity Increased COXIV protein levels; enhanced PGC-1α, NRF1, and TFAM gene expression and protein levels Attenuated loss of MMP; blocked cytochrome c release and caspase-3 activation; reduced the number of apoptotic cells Improved testicles ultrastructure; preserved sperm count and motility	Huang et al. (2023)
C57BL/6 mice testicles	LYC at 20 mg/kg.day^-1^ (oral administration) for 7 days prior to the exposure to X-ray irradiation (4 gy, 1 Gy/min)	Diminished MDA levels; enhanced SOD activity Prevented loss of MMP and ATP levels decline; restored complexes I, II, III, and IV activity; upregulated PGC-1α, NRF1, and TFAM protein levels	Qu et al. (2023)
​	​	Downregulated γ-H2X and bax protein levels; augmented Bcl-2 protein levels; decreased the number of apoptotic cells Failed to prevent sperm count decrease; improved sperm motility; reduced sperm abnormalities; reduced histopathological alterations	​
Porcine embryos	LYC at 0.1 µM for up to 6 days	Decreased ROS production; increased MMP levels; decreased cytochrome c release; reduced the number of apoptotic cells; attenuated SOD, CAT, and bax gene expression	Kang et al. (2021)
Mouse leydig TM3 cell lines	LYC at 20 µM to cells exposed to DBP at 200 µM for 24 h	Decreased ROS production; stimulated SOD activity Attenuated mitochondrial swelling, vacuolation, and cristae ablation; reduced abnormalities in mitochondrial autophagic vesicles; decreased LC3-II and Beclin1 protein levels; increased HSP60 and TOM20 protein levels Downregulated JAK2/STAT3 signaling pathway; diminished bax levels, cytochrome c release, and caspase-3 activation; conserved cell viability; reduced the number of apoptotic cells LYC induced similar effects when compared to JAK2/STAT3 inhibitor and NAC regarding cytoprotection in DBP-treated cells Stimulated testosterone synthesis	Wang et al. (2023b)
Grass carp kidney cells and grass carp kidney	*In vitro*: LYC at 1 µM in the presence of SMZ at 30 µM for 24 h *In vivo*: LYC at 10 mg/kg body weight, three times a day, to fish exposed to SMZ at 0.3 μg/L for 30 days	*In vitro* Reduced Beclin1, LC3-II, and ATG5 protein levels; in the presence of an inhibitor of autophagy (3-MA), LYC restored autophagy leading to apoptosis inhibition; attenuated loss of cell viability and apoptosis rate Upregulated Nrf2, HO-1, and NQO1; silencing of Nrf2 suppressed the effects on HO-1 and NQO1; reduced ROS production; silencing of Nrf2 suppressed the antioxidant and anti-apoptotic actions induced by LYC *In vivo* Attenuated mitochondrial swelling and nuclear atrophy; reduced number of apoptotic cells; decreased bax, caspase-9, and caspase-3 gene expression and protein levels; increased Bcl-2 gene expression and protein levels Increased GSH concentration and decreased MDA levels; restored Nrf2 nuclear levels; upregulated HO-1 and NQO1 Decreased GRP78, IRE1, ATF6, PERK, and CHOP gene expression and protein levels; downregulated eIF2α phosphorylation Reduced renal tubular dilation and epithelial abnormalities; reduced necrotic alterations	Zhao et al. (2025)

In neuronal systems, LYC mitigates oxidative and excitotoxic injury across diverse paradigms [including amyloid-β (Aβ), H_2_O_2_, trimethyltin (TMT), lead exposure, and 1-methyl-4-phenyl-1,2,3,6-tetrahydropyridine (MPTP)], consistently reducing mitochondrial and cytosolic ROS, preserving MMP, and limiting mPTP opening (Qu et al., 2011; Yi et al., 2013; Feng et al., 2016; Qu et al., 2016; Hwang et al., 2017; Huang et al., 2019; Qu et al., 2020). These events are accompanied by suppression of cytochrome c release, inhibition of caspase-9/-3 activation, downregulation of BCL2-associated X protein (Bax) and p53, and upregulation of B-cell lymphoma 2 (Bcl-2), collectively indicating stabilization of intrinsic apoptotic signaling (Hall-Younger and Tait, 2025). In most studies, attenuation of ROS precedes preservation of mitochondrial architecture, suggesting that LYC may primarily limit oxidative injury to redox-sensitive mitochondrial structures such as cardiolipin and ETC complexes, thereby secondarily reducing mPTP susceptibility. Alternatively, modulation of redox-sensitive signaling pathways, particularly NF-κB inhibition and inhibitor of κB (IκB) stabilization (Hwang et al., 2017), may shift transcriptional programs toward survival phenotypes that indirectly preserve mitochondrial performance. The reported upregulation of mitochondrial transcription factor A (TFAM), cytochrome c oxidase subunit 1 (COX1), and NADH:ubiquinone oxidoreductase core subunit 6 (ND6) (Yi et al., 2013; Qu et al., 2016; Hu et al., 2017) is frequently interpreted as evidence of mitochondrial biogenesis; however, without direct demonstration of mtDNA replication, nucleoid remodeling, or enhanced respiratory efficiency, these observations may instead represent adaptive transcriptional compensation secondary to oxidative stress (Qin et al., 2026). Thus, although neuronal models robustly demonstrate antioxidant and anti-apoptotic outcomes, they do not clarify whether LYC directly stabilizes mitochondrial structures, modulates, ETC electron flux, or primarily reshapes upstream redox signaling networks.

*In vivo* neurotoxicity models reinforce mitochondrial preservation but similarly lack functional resolution. In the 3-nitropropionic acid (3-NP) model, restoration of Complexes II, IV, and V activity together with recovery of thiol homeostasis (Sandhir et al., 2010) suggests improved coordination between electron transport and antioxidant defense systems. Nevertheless, in the absence of substrate-specific respiration analyses or high-resolution respirometry, it cannot yet be mechanistically prioritized whether LYC enhanced, ETC flux or simply prevented oxidative inhibition of respiratory complexes. Likewise, reductions in lipid peroxidation and restoration of glutathione (GSH)-dependent antioxidant systems in hippocampal and cortical tissues after colchicine exposure may reflect reinforcement of global redox buffering capacity rather than organelle-specific modulation (Prakash and Kumar, 2013). Following spinal cord injury (SCI), induction of mitochondrial biogenesis-associated genes such as cytochrome b and TFAM (Hu et al., 2017) further illustrates a recurrent limitation across the literature, namely the interpretation of transcriptional activation as functional mitochondrial remodeling in the absence of validation at the level of organelle abundance, ultrastructure, or bioenergetic recovery. Collectively, these studies highlight the importance of integrating transcriptional analyses with direct measurements of respiratory coupling, ATP synthesis efficiency, mitochondrial ROS generation, and ultrastructural remodeling.

Cardiac models subjected to ischemia/reperfusion (I/R) or hypoxia/reoxygenation (H/R) injury (Yue et al., 2012; Li et al., 2019) provide a more direct association between mitochondrial preservation and cell survival. In these systems, LYC inhibited mPTP opening, suppressed cytochrome c release, attenuated apoptotic protease activating factor-1 (APAF-1) activation, and reduced downstream caspase-9/-3 signaling, thereby limiting apoptotic progression. Importantly, the observation that atractyloside-mediated induction of mPTP opening abolished the anti-apoptotic effects of LYC (Li et al., 2019) strengthens the mechanistic association between mitochondrial permeability transition and cardioprotection. From a mechanistic perspective, mPTP regulation represents a convergence point for mitochondrial ROS, Ca^2+^ overload, and adenine nucleotide depletion (Hall-Younger and Tait, 2025). However, none of these upstream determinants were directly interrogated. Without assessing mitochondrial Ca^2+^ uptake kinetics, cyclophilin D activity, or redox-sensitive thiol modifications within pore components, it remains unclear whether LYC directly modulated pore opening probability or indirectly reduced triggering stimuli. Furthermore, the contribution of ETC-derived ROS bursts during reperfusion, a critical driver of mPTP activation, was not quantitatively addressed, leaving unresolved whether LYC acts by dampening electron leak at specific, ETC sites (e.g., Complex I or III) or by scavenging ROS after their formation.

More recently, emerging evidence has expanded the mechanistic scope of LYC-mediated cardioprotection beyond canonical apoptosis toward mitochondria-associated inflammatory cell death pathways. In cardiac microvascular endothelial cells and rat hearts subjected to H/R injury, Sha et al. (2026) demonstrated that LYC suppressed the YTH N^6^-methyladenosine RNA binding protein 1/E2F transcription factor 8/fatty acid binding protein 3 (YTHDF1/E2F8/FABP3, respectively) axis, reduced cytosolic mtDNA accumulation, inhibited cyclic GMP-AMP synthase/stimulator of interferon genes (cGAS-STING, respectively) signaling, and attenuated pyroptosis-associated markers including cleaved caspase-1 and gasdermin D N-terminal domain (GSDMD-terminal). These effects were accompanied by restoration of ATP levels, enhanced nitric oxide (NO) production, increased platelet endothelial cell adhesion molecule-1 (CD31) and phosphorylated-endothelial nitric oxide synthase (eNOS) levels, and suppression of interleukin-1β (IL-1β), interleukin-6 (IL-6), and interleukin-18 (IL-18). Importantly, silencing of FABP3 or YTHDF1 reproduced several protective effects induced by LYC, whereas overexpression of E2F8 or FABP3 abolished them, providing stronger causal support than most studies currently available in the field. Mechanistically, these findings suggest that preservation of mitochondrial integrity may limit mtDNA leakage and thereby attenuate mitochondria-dependent inflammatory signaling. Nevertheless, whether LYC directly stabilizes mitochondrial membranes or indirectly reduces mtDNA release through broader metabolic preservation remains unresolved. Furthermore, although molecular docking analyses suggested potential interaction between LYC and YTHDF1, biochemical validation under physiologically relevant conditions is still lacking.

In hepatic and pancreatic systems, LYC appears to modulate both mitochondrial and extramitochondrial ROS-generating pathways, positioning it as a broader coordinator of cellular redox homeostasis. In cytochrome P450 family 2 subfamily E member 1 (CYP2E1)-overexpressing HepG2 cells, attenuation of ethanol-induced apoptosis was associated with increased mitochondrial glutathione levels and reduced oxidative burden (Xu et al., 2003), suggesting that suppression of ROS generation may precede mitochondrial preservation. Similarly, inhibition of NADPH oxidase activity and NF-κB signaling in pancreatic acinar cells (Lee et al., 2021) indicates that cytosolic ROS-producing systems substantially contribute to mitochondrial dysfunction. In this context, preservation of MMP and ATP levels may represent downstream consequences of reduced oxidative stress rather than direct modulation of mitochondrial bioenergetics. In aflatoxin B1 (AFB1)-induced toxicity, maintenance of Bcl-2 levels together with suppression of cytochrome c release and caspase activation (Xu et al., 2019) further supports anti-apoptotic signaling, although the extent to which mitochondrial outer membrane permeabilization itself was specifically targeted remains uncertain. Collectively, these findings suggest that LYC operates within an integrated redox network spanning mitochondrial, cytosolic, and microsomal compartments, complicating identification of its dominant mechanistic layer.

Recent studies have also identified cuproptosis as a potential target of LYC-mediated mitochondrial protection. In chicken heart exposed to atrazine (ATZ), Li et al. (2025b) demonstrated that LYC attenuated mitochondrial cristae fragmentation, restored tricarboxylic acid (TCA) cycle intermediates, reduced copper accumulation, and downregulated multiple cuproptosis-related regulators, including copper transporter receptor (CTR1), ferredoxin 1 (FDX1), lipoic acid synthetase (LIAS), dihydrolipoamide S-acetyltransferase (DLAT), cytochrome c oxidase copper chaperone (COX17), and ATPase copper transporting α (ATP7A). Notably, suppression of lipoylated-DLAT accumulation, together with restoration of reduced nicotinamide adenine dinucleotide (NADH) levels and mitochondrial membrane integrity, suggests that LYC may interfere with copper-dependent proteotoxic stress linked to destabilization of Fe-S cluster proteins and collapse of TCA cycle metabolism. In parallel, LYC attenuated markers of cellular senescence and suppressed senescence-associated secretory phenotype (SASP)-related inflammatory mediators, indicating that modulation of mitochondrial metabolism may extend beyond cell death regulation toward broader stress adaptation programs (Victorelli et al., 2023; Marzetti et al., 2024). However, because pharmacological inhibition or genetic manipulation of cuproptosis-associated proteins was not performed, it remains uncertain whether suppression of cuproptosis represented a primary mechanism underlying cytoprotection or a downstream consequence of generalized mitochondrial stabilization. Moreover, the relationship between restoration of TCA cycle homeostasis, attenuation of inflammatory signaling, and suppression of senescence remains mechanistically unresolved.

A related framework emerged in LMH hepatocytes and chicken liver exposed to atrazine, where Li et al. (2026) demonstrated that LYC restored pyruvate and α-ketoglutarate levels, reduced copper accumulation, attenuated mitochondrial ultrastructural damage, and suppressed expression of cuproptosis-associated proteins including CTR1, FDX1, LIAS, DLAT, COX17, ATP7B, and antioxidant 1 copper chaperone (ATOX1). Importantly, ATOX1 knockdown abolished cuproptosis in ATZ-treated hepatocytes, supporting a mechanistic role for copper trafficking in mitochondrial injury. Molecular docking analyses further suggested direct interaction between LYC and ATOX1, raising the possibility that LYC modulates mitochondrial copper homeostasis through interaction with copper chaperone systems. Nevertheless, because mitochondrial respiration, Fe-S cluster stability, and proteotoxic stress responses were not interrogated at the functional level, the extent to which LYC specifically inhibits cuproptosis rather than broadly preserving mitochondrial homeostasis remains incompletely defined. The convergence between restoration of antioxidant defenses, attenuation of inflammatory injury, and normalization of mitochondrial metabolism further supports the possibility that suppression of cuproptosis may emerge secondarily from integrated stabilization of mitochondrial stress responses (Tian et al., 2023).

Reproductive and developmental models extend this framework by linking mitochondrial bioenergetics to endocrine, reproductive, and developmental outcomes. LYC-induced increases in dehydrogenase activity [including succinate dehydrogenase (SDH), malate dehydrogenase (MDH), and isocitrate dehydrogenase (IDH)] and ETC complex activity, together with upregulation of PGC-1α, nuclear respiratory factor 1 (NRF1), and TFAM (Aly et al., 2012; Kang et al., 2021; Huang et al., 2023; Qu et al., 2023; Wang et al., 2023b), are consistent with enhanced mitochondrial capacity; however, these conclusions are not supported by direct measurements of oxidative phosphorylation efficiency or expansion of mitochondrial networks. Importantly, it was not investigated whether PGC-1α-dependent signaling contributed directly to the antioxidant and anti-apoptotic effects promoted by LYC (Aquilano et al., 2013; Abu Shelbayeh et al., 2023). The reported inhibition of Janus kinase 2 (JAK2)/STAT3 signaling and modulation of mitophagy in Leydig cells (Wang et al., 2023b) further suggests regulation of mitochondrial turnover, yet mitophagic flux [e.g., PTEN-induced putative kinase 1 (PINK1)/Parkin recruitment, microtubule-associated protein 1 light chain 3 (LC3) turnover in mitochondria-specific contexts] was not quantified. Consequently, it remains unclear whether LYC prevented excessive mitochondrial degradation under stress conditions or directly remodeled mitochondrial turnover pathways (Wang et al., 2026). In embryonic systems, maintenance of MMP and attenuation of apoptotic signaling similarly indicate preservation of mitochondrial function, but the underlying drivers (whether related to redox buffering, metabolic adaptation, or developmental signaling modulation) remain incompletely resolved (Kang et al., 2021).

Aquatic and environmental toxicology models provide some of the strongest evidence supporting the involvement of redox-responsive transcriptional programs in LYC-mediated cytoprotection. In sulfamethoxazole-exposed grass carp kidney cells and tissues, Zhao et al. (2025) demonstrated that activation of the Nrf2/HO-1/NAD(P)H quinone dehydrogenase 1 (NQO1) axis was required for antioxidant and anti-apoptotic protection, as Nrf2 silencing abolished several beneficial effects induced by LYC. Concurrent suppression of ROS accumulation, ER stress signaling, apoptosis, and autophagy-related markers suggests preservation of mitochondrial resilience within a broader adaptive stress-response network. However, because autophagic flux and mitochondria-specific turnover pathways were not dynamically assessed, it remains difficult to distinguish selective inhibition of excessive mitophagy from broader suppression of autophagic signaling (Wang et al., 2026). These findings therefore reinforce a recurring challenge across the field: differentiating direct modulation of mitochondrial quality-control mechanisms from secondary normalization of stress-responsive pathways following restoration of cellular redox balance.

Across experimental systems, a central limitation persists: mitochondrial function is frequently inferred from surrogate biochemical or transcriptional markers rather than directly quantified using integrated high-resolution methodologies. Critical parameters (including site-specific ROS generation within the, ETC., coupling efficiency between electron transport and ATP synthesis, proton leak kinetics, mitochondrial substrate utilization, and dynamic regulation of mitochondrial turnover) remain insufficiently explored. Moreover, the absence of pharmacokinetic and subcellular distribution analyses prevents determination of whether LYC accumulates within mitochondrial membranes at concentrations sufficient to directly modulate cardiolipin domains, ETC complexes, copper-binding proteins, or redox-sensitive signaling platforms. The potential hormetic nature of LYC-mediated redox modulation, whereby mild oxidative cues trigger adaptive mitochondrial responses, also warrants systematic investigation.

Taken together, current evidence supports a model in which LYC modulates mitochondrial redox biology and mitochondria-related cell death through multilayered mechanisms involving ROS suppression, stabilization of mitochondrial architecture, transcriptional reprogramming, inflammatory signaling modulation, and preservation of apoptotic checkpoints. Emerging evidence further suggests that these effects may extend beyond canonical apoptosis to include regulation of pyroptosis, cuproptosis, and mitochondrial danger-associated signaling pathways. However, the predominance of associative evidence continues to limit establishment of mechanistic hierarchy. LYC may function simultaneously as a redox buffer, a regulator of stress-responsive transcriptional programs such as Nrf2 and NF-κB, a suppressor of mitochondria-associated inflammatory signaling, and an indirect stabilizer of mitochondrial homeostasis, rather than as a classical mitochondria-targeted compound with clearly defined molecular targets. Resolving this ambiguity will require integration of quantitative bioenergetics, compartment-specific redox analyses, multi-omics profiling, pharmacokinetic-pharmacodynamic characterization, and targeted genetic or pharmacological perturbation of candidate mitochondrial pathways. Only through such mechanistically resolved and spatially integrated investigations will it become possible to determine whether LYC should be classified as a direct mitochondrial modulator or as a broader systems-level regulator of mitochondrial stress adaptation.

### 2.3 Effects of LYC on mitochondrial biogenesis

Accumulating evidence indicates that LYC modulates signaling networks canonically associated with mitochondrial biogenesis; however, the prevailing interpretation that LYC induces *bona fide* mitochondrial biogenesis remains only partially supported when evaluated against the structural and functional criteria required to define *de novo* organelle expansion (Figure 1; Table 3). Across experimental systems, LYC consistently activates the AMPK/SIRT1/PGC-1α axis and increases expression of nuclear-encoded mitochondrial genes and OXPHOS subunits, yet these molecular signatures alone do not demonstrate coordinated mtDNA replication, organelle expansion, and functional integration. Instead, they may reflect redox-sensitive transcriptional reprogramming that preserves resident mitochondrial populations, enhances respiratory efficiency, or attenuates stress-induced mitochondrial loss (Liu et al., 2023). Consequently, a central unresolved issue emerges across the field: whether LYC functions primarily as an inducer of canonical organelle biogenesis, a facilitator of mitochondrial fitness under stress conditions, or a systemic metabolic regulator whose mitochondrial effects arise secondarily from broader adaptive remodeling.

**TABLE 3:** The effects of LYC on mitochondrial biogenesis.

Biological target	Experimental model	Major findings	References
C57BL/6J mice cerebral cortex, hippocampus, and liver	LYC at 0.03% w/w (added to standard chow) for 5 weeks prior to the administration of LPS at 0.25 mg/kg.day^-1^ (i.p. injection) for additional 9 days	Cerebral cortex and hippocampus Stimulated SIRT1, PGC-1α, COX5B, COX7A1, COX8B, and CYCS gene expression; increased complexes I, II, III, and IV protein levels Stimulated BDNF, NGF, and NT3, and NT4 gene expression; increased SNAP-25 and PSD-95 protein levels; increased protein levels of phosphorylated IRS-1^T612^ and decreased IRS-1^S307^ protein levels; prevented Akt and GSK3β inhibition; stimulated GLUT1, GLUT3, and GLUT4 gene expression; reduced PTP1B protein levels; prevented histopathological abnormalities Attenuated JNK, ERK, and p38 activation; decreased NF-κB, iNOS, and COX-2 protein levels; reduced TNF-α, IL-1β, and IL-6 gene expression Enhanced HO-1 and NQO1 protein levels Liver Stimulated IRS-1/Akt/GSK3β axis; decreased PTP1B protein levels; increased GLUT2 gene expression and protein levels Augmented SIRT1, PGC-1α, and complexes I – IV protein levels; stimulated SIRT1, PGC-1α, COX7A1, COX8B, and CYCS gene expression Induced anti-inflammatory effects (in a similar fashion when compared to mice cerebral cortex and hippocampus)	Wang et al. (2019)
C57BL/6 mice kidney	LYC at 20 mg/kg (oral administration) 24 h prior to the exposure to LPS at 10 mg/kg (i.p. injection) for additional 24 h	Stimulated PGC-1α gene expression Enhanced Nrf2 gene expression and protein levels; stimulated SOD, GPX, and CAT activity; reduced MDA levels and ROS production; prevented loss of MMP Downregulated TLR4 and NF-κB gene expression; decreased NF-κB, TLR4, TNF-α, and IL-6 protein levels; diminished MPO activity Improved renal function	Salari et al. (2022)
HepG2 cells and CD-1 mice liver	*In vitro* LYC at 50 µM for 8 h before administration of H_2_O_2_ at 200 µM for additional 24 h *In vivo* LYC at 0.03% (w/w, added to the standard chow) during the exposure to D-gal at 150 mg/kg.day^-1^ (i.p. injected) for 8 weeks	*In vitro* Attenuated COX-2 and IL-1β protein levels; increased NQO-1 protein levels Reduced the protein levels of phosphorylated IRS-1^S307^; stimulated phosphorylation of Akt^S437^ and GSK-3β; enhanced PPARα and FGF21 protein levels Stimulated phosphorylation of AMPK; increased PGC-1α and SIRT1 protein levels; prevented loss of MMP Silencing of FGF21 suppressed the effects LYC induced on AMPK, PGC-1α, SIRT1, IRS-1, Akt, GSK3β, and MMP levels	Wang et al. (2022b)
​	​	Oligomycin suppressed the effects LYC induced on IRS-1, Akt, GSK3β, and MMP levels *In vivo* Stimulated phosphorylation of AMPK; increased PGC-1α and SIRT1 protein levels; enhanced PPARα and FGF21 protein levels Enhanced GSH levels; stimulated SOD and CAT activity Reduced the protein levels of phosphorylated IRS-1^S307^; stimulated phosphorylation of AKT^S437^ and GSK-3β	​
CD-1 mice liver and kidney	LYC at 0.03% w/w (added to the standard chow) during the exposure to D-gal at 150 mg/kg.day^-1^ (i.p. injected) for 8 weeks	Upregulated AMPK, SIRT1, and PGC-1α; increased COX5a, NDUFS1, NDUFS2, NDUFS3 protein level Increased Nrf2, HO-1, and NQO-1 protein levels Suppressed JNK, ERK, and p38 activation; blocked NF-κB activation; decreased IL-1β, TNF-α, and COX-2 protein levels Restored insulin signaling	Wang et al. (2022d)

In lipopolysaccharide (LPS)-challenged mice, LYC restored SIRT1 and PGC-1α expression while increasing levels of respiratory complex subunits and mitochondrial-associated genes such as cytochrome c oxidase subunit 5B (mitochondrial - COX5B), cytochrome c oxidase subunit 7A1 (mitochondrial - COX7A1), cytochrome c oxidase subunit 8B (mitochondrial - COX8B), and cytochrome c (somatic - CYCS) (Wang et al., 2019). Within the canonical biogenesis framework, SIRT1-mediated deacetylation of PGC-1α would be expected to enhance NRF1/nuclear respiratory factor 2 (NRF2)-dependent transcriptional programs and TFAM-mediated mtDNA replication (Liu et al., 2023). Importantly, however, TFAM activity, nucleoid remodeling, and mtDNA expansion were not directly examined, leaving the proposed biogenic cascade lacking mechanistic closure. An alternative interpretation is that suppression of NF-κB signaling and inflammatory mediators primarily relieved inflammation-associated repression of mitochondrial gene expression, thereby restoring mitochondrial protein abundance without necessarily increasing organelle number (Marchi et al., 2023). This interpretation is reinforced by the concomitant activation of insulin signaling [insulin receptor substrate-1(IRS-1)/Akt/glycogen synthase kinase 3β (GSK3β)], glucose transport pathways (glucose transporters 1,3, and 4 - GLUT1/3/4, respectively), and neurotrophic signaling involving brain-derived neurotrophic factor (BDNF) and nerve growth factor (NGF), which collectively suggest broader neuro-metabolic remodeling capable of secondarily increasing mitochondrial demand (Sayehmiri et al., 2024). In this context, elevated OXPHOS subunit expression may reflect energetic recalibration rather than autonomous organelle biogenesis. The parallel hepatic response observed in the same model additionally raises the possibility of inter-organ metabolic communication, potentially mediated by circulating factors such as FGF21, although this axis was not experimentally interrogated (Prida et al., 2022). Without quantitative morphometric analyses or demonstration of increased respiratory capacity coupled to organelle expansion, interpretation as canonical mitochondrial biogenesis remains provisional.

A similarly complex scenario emerges in sepsis-associated acute kidney injury. In this model, LYC increased PGC-1α expression, activated Nrf2 signaling, restored antioxidant defenses, and preserved mitochondrial membrane potential (Salari et al., 2022). Mechanistically, coactivation of PGC-1α and Nrf2 suggests integration between mitochondrial remodeling and redox adaptation (Gureev et al., 2019). Nevertheless, because Nrf2 primarily regulates antioxidant and detoxification programs rather than core mtDNA replication machinery, its contribution to mitochondrial remodeling is likely indirect, mediated through preservation of redox conditions permissive for mitochondrial function (Holmström et al., 2016). Accordingly, increased PGC-1α expression in this context may represent adaptive bioenergetic reprogramming secondary to attenuation of oxidative injury to mtDNA, cardiolipin, and ETC complexes rather than initiation of a complete biogenic program (Gureev et al., 2019; Cao et al., 2025). Importantly, the absence of organelle-level validation (including quantitative morphometry, mtDNA assessment, and substrate-specific respiratory analyses) prevents differentiation between increased mitochondrial abundance and improved performance of pre-existing organelles. Likewise, because neither PGC-1α nor Nrf2 signaling was experimentally perturbed (e.g., genetic knockdown or pharmacological inhibition), causal hierarchy remains undefined, and these pathways may represent associative markers of improved cellular redox status rather than primary mediators of nephroprotection. An additional unresolved dimension involves the lack of pharmacokinetic and subcellular localization data, which obscures whether LYC reaches mitochondrial compartments at concentrations sufficient to directly influence biogenic signaling machinery.

In hepatic and hepatocyte aging models, LYC activated AMPK, SIRT1, PGC-1α, PPARα, and FGF21 signaling while attenuating mitochondrial dysfunction and senescence-associated phenotypes (Wang et al., 2022b). This signaling constellation suggests a multi-tiered regulatory network linking energy sensing (AMPK), deacetylation (SIRT1), transcriptional coactivation (PGC-1α), and endocrine signaling (FGF21/PPARα), probably leading to metabolic adaptation. Compared with other studies, silencing of FGF21 provided stronger mechanistic support for pathway involvement, indicating that endocrine signaling contributes directly to mitochondrial adaptation and systemic metabolic rewiring (Prida et al., 2022). Mechanistically, FGF21 may enhance mitochondrial oxidative metabolism through PPARs-associated transcriptional programs (Vernia et al., 2014); however, its direct contribution to mtDNA replication, mitochondrial expansion, or respiratory supercomplex assembly was not experimentally resolved. The use of oligomycin further demonstrated that intact ATP synthesis was required for the signaling effects induced by LYC, suggesting close coupling between mitochondrial energetic status and downstream metabolic responses. Nevertheless, this observation does not establish whether LYC increased mitochondrial number, improved ATP synthesis efficiency, or both. Importantly, integration of IRS-1/PI3K/Akt/GSK3β signaling further suggests that mitochondrial remodeling may occur in parallel with enhanced metabolic substrate handling, raising the possibility that improved mitochondrial parameters reflect metabolic rewiring rather than intrinsic biogenic activation. As in other systems, the absence of direct structural confirmation prevents definitive mechanistic interpretation.

Additional observations in liver and kidney tissues demonstrated concurrent activation of Nrf2 and AMPK/SIRT1/PGC-1α pathways together with increased expression of OXPHOS-associated proteins (Wang et al., 2022c). Although these findings reinforce a recurring mechanistic pattern, they also highlight a persistent conceptual ambiguity. Nrf2-dependent antioxidant adaptation and AMPK/SIRT1-mediated energetic sensing may converge to preserve mitochondrial function without necessarily driving organelle expansion (Esteras and Abramov, 2022; Luchkova et al., 2024). Because Nrf2 does not directly regulate the core machinery responsible for mtDNA replication, its influence on mitochondrial remodeling likely depends on maintenance of redox conditions compatible with, ETC function and mitochondrial gene expression (Holmström et al., 2016). Furthermore, the directional relationship between mitochondrial adaptation and inflammatory resolution remains insufficiently delineated. Reduced mitochondrial ROS production and diminished release of damage-associated molecular patterns may attenuate inflammation; conversely, suppression of inflammatory signaling may restore mitochondrial transcriptional competence (Yu et al., 2025). Without temporal and mechanistic dissection, these processes remain associative in nature.

Collectively, the current literature is limited by a persistent reliance on transcriptional and protein-level surrogates of mitochondrial biogenesis without integrated assessment of structural (mtDNA copy number, mitochondrial density, cristae architecture, organelle number), functional (respiratory capacity, coupling efficiency), and dynamic (fusion/fission balance, mitophagic flux) mitochondrial parameters. Consequently, differentiation between increased mitochondrial content, enhanced respiratory efficiency, and reduced mitochondrial turnover remains difficult. For example, attenuation of mitophagy (suggested indirectly in some contexts) could increase apparent mitochondrial abundance without genuine organelle biogenesis, whereas improved, ETC efficiency could elevate ATP production independently of changes in mitochondrial number (Gureev et al., 2019). Likewise, variability in LYC dosing, exposure duration, tissue-specific accumulation, and metabolic context complicates mechanistic interpretation, particularly in the absence of direct analyses of intramitochondrial localization and interactions with membrane lipids or protein complexes. Another major limitation involves the lack of spatially and temporally resolved analyses capable of distinguishing acute stress adaptation from sustained mitochondrial remodeling.

Taken together, current evidence positions LYC as a promising modulator of interconnected signaling networks associated with mitochondrial adaptation, including AMPK/SIRT1/PGC-1α, FGF21/PPAR, and Nrf2-associated pathways. Through these systems, LYC appears to integrate redox regulation, energetic sensing, and systemic metabolic communication with preservation of mitochondrial fitness. However, definitive evidence demonstrating canonical mitochondrial biogenesis (defined by coordinated mtDNA replication, organelle expansion, and stable functional integration) remains insufficient. At present, LYC cannot be conclusively classified as a true inducer of mitochondrial biogenesis, because most studies rely predominantly on indirect molecular markers without comprehensive structural and functional validation. Instead, the available evidence suggests that LYC may operate primarily as a facilitator of mitochondrial resilience and metabolic adaptation under pathological stress conditions. Resolving this mechanistic hierarchy will require rigorously integrated experimental approaches combining quantitative morphometry, high-resolution bioenergetics, mtDNA dynamics, real-time imaging, pharmacokinetic characterization, and targeted perturbation of candidate signaling pathways. Only through such causality-driven and spatially resolved investigations will it become possible to determine whether LYC genuinely promotes canonical organelle biogenesis or instead preserves mitochondrial homeostasis through broader metabolic and redox reprogramming.

### 2.4 Effects of LYC on mitochondrial fusion and fission

**FIGURE 3:**
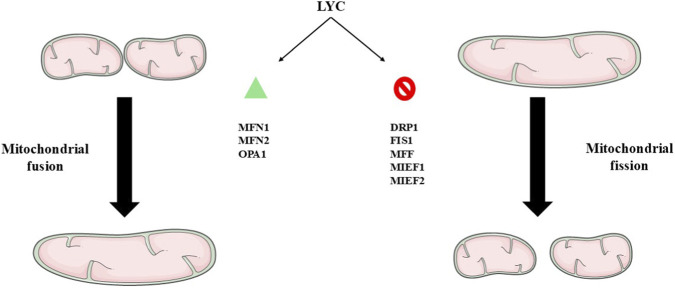
A summary of the effects promoted by LYC on mitochondrial fusion and fission. Although research in this area is scarce, there is evidence that LYC stimulates mitochondrial fusion and represses mitochondrial fission. LYC upregulated MFN1, MFN2, and OPA1, key proteins in the modulation of mitochondrial fusion. On the other hand, this carotenoid downregulated DRP1, FIS1, MFF, MIEF1, and MIEF2 proteins and inhibited mitochondrial fission, possibly playing a role in attenuating disorders in the interaction between mitochondrial and ER membranes. Mechanistically, data demonstrating how LYC induces these effects are lacking. Therefore, further investigation is needed. Please, read the text and analyze the tables for more detailed information. This figure exhibits an image created by Servier Medical Art, which is licensed under a Creative Commons Attribution 4.0 Unported License (https://creativecommons.org/licenses/by/4.0/).

LYC has been consistently associated with preservation of mitochondrial reticular organization, typically characterized by reduced fragmentation and maintenance of fusion-permissive network connectivity under conditions of cellular stress (Figure 3; Table 4). Nevertheless, the mechanistic basis underlying these effects remains incompletely resolved because most available evidence derives from surrogate molecular endpoints rather than dynamically resolved analyses of mitochondrial behavior. Canonically, mitochondrial dynamics are coordinated through the balanced activities of outer membrane GTPases [mitofusin 1 (MFN1) and MFN2], inner membrane remodeling proteins such as optic atrophy 1 (OPA1), and fission machinery centered on dynamin-related protein 1 (DRP1) recruitment and oligomerization at constriction sites (Xu et al., 2024; Wang et al., 2026). Although LYC modulates expression of these regulators across multiple models, it remains uncertain whether it directly controls their activation state, subcellular distribution, or membrane interactions, or instead generates intracellular conditions permissive for mitochondrial connectivity through broader modulation of redox balance, Ca^2+^ handling, membrane composition, and inter-organelle communication. Clarifying this mechanistic hierarchy is essential for defining whether LYC functions as a direct regulator of mitochondrial network remodeling or as a broader cellular stress-adaptation regulator that secondarily preserves mitochondrial architecture.

**TABLE 4:** The effects of LYC on mitochondrial fusion and fission.

Biological target	Experimental model	Major findings	References
ICR mice liver	LYC at 5 mg/kg.day^-1^ (body weight) during the exposure to DEHP at 500 or 1000 mg/kg.day^-1^ for 4 weeks (oral administration)	Alleviated mitochondrial swelling; reduced the number of blurry mitochondrial membranes Upregulated MFN1, MFN2, and OPA1 mRNA levels; decreased MIEF1, MIEF2, DRP1, FIS1, and MFF gene expression; reduced DRP1 protein levels; increased OPA1, MFN1, and MFN2 protein levels Downregulated VDAC1 and GRP75 protein levels; mitigated ER stress (reduced protein levels of GRP78, ATF6, and ATF4); alleviated ER-mitochondria systems abnormalities	Zhao et al. (2021)
ICR mice liver	LYC at 10 mg/kg.day^-1^ (intragastrically administrated) during the exposure to a combination of ZEN (10 mg/kg.day^-1^), DON (1 mg/kg.day^-1^), and AFB_1_ (0.5 mg/kg.day^-1^) (i.p. injected) for 14 days	Improved total antioxidant capacity; reduced mitochondrial swelling and vacuolization Suppressed the toxins-induced upregulation of SIRT1, TFAM, PGC-1α, NRF1, and NRF2 gene expression (mRNA levels); downregulated MFN1, DRP1, and FIS1 gene expression; did not change MFN2 and OPA1 gene expression; upregulated MFN1 protein levels; decreased MFN2 protein levels; downregulated DRP1 protein levels; decreased CYP2E1 protein levels Attenuated hepatotoxicity and liver dysfunction; alleviated hepatic fibrosis	Lin et al. (2023)

In di-(2-ethylhexyl) phthalate (DEHP)-exposed mice, LYC restored mitochondrial ultrastructure while shifting the molecular profile toward a fusion-associated state characterized by increased MFN1, MFN2, and OPA1 together with reduced DRP1, mitochondrial fission 1 protein (FIS1), and mitochondrial fission factor (MFF) expression (Zhao et al., 2021). Within the canonical framework of mitochondrial dynamics, such changes would be expected to promote outer membrane tethering, stabilization of cristae organization, and suppression of DRP1-mediated membrane scission (Wang et al., 2026). Importantly, however, expression levels alone provide limited insight into the functional status of these proteins, which is critically governed by post-translational regulation, proteolytic processing, and mitochondrial recruitment dynamics (Xu et al., 2024). Because DRP1 phosphorylation state, OPA1 isoform balance, and protein localization were not examined, it remains difficult to determine whether LYC truly altered the operational state of the fusion-fission machinery. Mechanistically, an alternative explanation emerges from the concomitant attenuation of ER stress and normalization of MAMs. Given that MAMs regulate Ca^2+^ transfer, lipid exchange, and DRP1 recruitment, stabilization of ER-mitochondria coupling could indirectly reduce fission permissiveness by limiting Ca^2+^-dependent calcineurin activation and subsequent DRP1 translocation (Mohan and Talwar, 2025). The parallel downregulation of voltage-dependent anion channel 1 (VDAC1) and heat shock protein family A member 9 (GRP75) further supports modulation of inter-organelle tethering complexes as a potential upstream event. In this context, preservation of mitochondrial connectivity may reflect attenuation of oxidative and Ca^2+^-dependent stress signaling rather than direct induction of fusion itself (Adebayo et al., 2021). Without time-resolved analyses of mitochondrial morphology or targeted perturbation of DRP1/MFN function, causal interpretation remains incomplete.

A more complex regulatory profile emerged in the mixed-mycotoxin model, in which LYC attenuated mitochondrial structural disruption and reduced DRP1 and FIS1 abundance while simultaneously generating discordant mRNA and protein profiles for MFN1, MFN2, and OPA1 (Lin et al., 2023). This divergence strongly suggests regulation beyond transcriptional control, potentially involving altered protein turnover, mitochondrial import efficiency, ubiquitin-dependent degradation, or proteolytic processing of OPA1 by stress-responsive proteases such as OMA1 zinc metallopeptidase (OMA1) and ATP-dependent zinc metalloprotease YME1L1 (YME1L) (Anand et al., 2014). Notably, the reduction in MFN2 protein abundance despite preserved transcript levels challenges the interpretation of a uniformly fusion-dominant phenotype and instead supports the possibility that mitochondrial network remodeling is selectively recalibrated rather than globally shifted (Adebayo et al., 2021). Similarly, because DRP1 functionality depends strongly on activation state and mitochondrial recruitment dynamics, reduced total protein abundance does not necessarily indicate suppression of fission if the remaining DRP1 pool is preferentially activated or enriched at mitochondrial constriction sites (Adaniya et al., 2019). An additional layer of complexity emerges from the concurrent suppression of CYP2E1, which may attenuate ROS-dependent signaling pathways linked to DRP1 activation and mitochondrial fragmentation (Pedrera et al., 2025). Furthermore, the simultaneous downregulation of mitochondrial biogenesis-associated genes such as PGC-1α, NRF1, and TFAM alongside modulation of fusion/fission regulators indicates that mitochondrial remodeling and biogenesis become partially uncoupled in this context. Collectively, these findings support the interpretation that LYC may preferentially preserve mitochondrial ultrastructural continuity and network stability rather than stimulate organelle renewal.

Across both models, a central limitation remains the absence of direct and quantitative assessment of mitochondrial network plasticity. Fusion and fission are intrinsically dynamic phenomena that require time-resolved evaluation, yet current studies rely predominantly on steady-state molecular analyses. Consequently, it remains uncertain whether LYC genuinely alters mitochondrial dynamics or instead preserves network morphology under conditions of reduced cellular stress. Moreover, because pharmacological or genetic perturbation of key regulators such as DRP1 and MFN1/2 was not performed, it is not possible to determine whether modulation of fusion-fission balance is mechanistically required for the protective effects attributed to LYC. These limitations leave open the possibility that altered mitochondrial dynamics represent downstream consequences of broader stress adaptation rather than primary mediators of cytoprotection.

Another mechanistically important but insufficiently explored dimension involves mitochondrial membrane biophysics. Fusion and fission are highly sensitive to membrane lipid composition, particularly cardiolipin content, membrane curvature, and lipid peroxidation state (Paradies et al., 2019). As a lipophilic carotenoid, LYC may theoretically incorporate into mitochondrial membranes and influence membrane packing properties, susceptibility to oxidative damage, and cristae organization. Such effects could indirectly modulate OPA1-dependent inner membrane remodeling and DRP1-mediated constriction independently of transcriptional regulation. Likewise, redox-sensitive modifications of fusion/fission proteins, including thiol oxidation, may be altered by the antioxidant properties of LYC, further complicating discrimination between direct and indirect regulatory mechanisms. However, because no studies have directly examined intramitochondrial localization of LYC, membrane lipid interactions, or biophysical alterations in mitochondrial membranes, this potentially central mechanistic layer remains largely unexplored.

In conclusion, current evidence indicates that LYC preserves mitochondrial network organization under toxic and metabolic stress conditions through mechanisms that likely extend beyond direct regulation of canonical fusion-fission machinery. Rather than functioning exclusively as a mitochondria-targeted modulator of DRP1-, MFN-, or OPA1-dependent dynamics, LYC appears to reshape the intracellular environment toward fusion-permissive states by integrating redox homeostasis, ER–mitochondria communication, Ca^2+^ handling, membrane biophysics, and stress-response signaling. Within this framework, attenuation of oxidative burden, stabilization of MAMs, and preservation of membrane structural properties may collectively reduce stress-induced fragmentation while maintaining mitochondrial connectivity, cristae continuity, and network plasticity. Importantly, the available data further suggest that mitochondrial dynamics may become partially uncoupled from canonical mitochondrial biogenesis pathways under certain pathological contexts, reinforcing the complexity of mitochondrial remodeling induced by LYC. However, the predominance of steady-state molecular signatures and endpoint analyses continues to limit establishment of mechanistic hierarchy and causal interpretation. Consequently, it remains unresolved whether modulation of mitochondrial dynamics represents a primary mechanism underlying the cytoprotective effects of LYC or instead reflects secondary preservation of mitochondrial architecture following broader attenuation of cellular stress.

Future progress in this field will require integrated approaches combining quantitative live-cell imaging of mitochondrial network plasticity with mechanistic interrogation of the core fusion–fission machinery. Spatially resolved analyses of DRP1 activation, OPA1 processing, mitochondrial recruitment dynamics, and MAM organization will be particularly important for establishing causal hierarchy. Likewise, integration of super-resolution imaging, quantitative morphometry, lipidomics, and targeted manipulation of mitochondrial dynamics regulators will be essential for determining whether LYC acts primarily through direct organelle-level remodeling or through broader regulation of cellular stress adaptation. Extending these analyses to human hepatocytes and metabolically relevant disease models may further clarify whether LYC can function as a physiologically relevant modulator of mitochondrial network architecture rather than merely an indirect antioxidant stabilizer.

### 2.5 Effects of LYC on mitophagy

**FIGURE 4:**
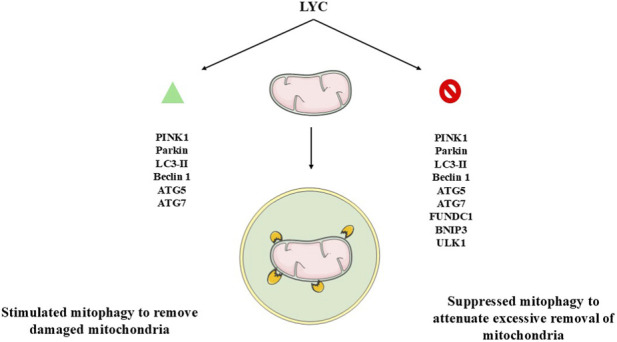
A summary of the effects promoted by LYC on mitophagy. Depending on the context, LYC stimulated or suppressed mitophagy. Through the regulation of PINK1 and Parkin, among other proteins, LYC promoted mitophagy by stimulating the removal of damaged mitochondria. This led to beneficial effects in cells exposed to different stress situations. On the other hand, LYC blocked mitophagy under other conditions, preventing excessive mitochondrial degradation. Mechanistically, data demonstrating how LYC induces these effects are lacking. Therefore, further investigation is needed. Please, read the text and analyze the tables for more detailed information. This figure exhibits an image created by Servier Medical Art, which is licensed under a Creative Commons Attribution 4.0 Unported License (https://creativecommons.org/licenses/by/4.0/).

LYC has increasingly emerged as a context-dependent regulator of mitophagy; however, the mechanistic basis of this regulation remains incompletely resolved (Figure 4; Table 5). Canonically, mitophagy is initiated by mitochondrial depolarization, oxidative injury, or mtDNA instability, which stabilize PINK1 on the outer mitochondrial membrane and promote Parkin recruitment, ubiquitination of mitochondrial substrates, and engagement of autophagic adaptors such as sequestosome 1 (SQSTM1/p62), optineurin (OPTN), and nuclear domain 10 protein 52 (NDP52), culminating in lysosomal degradation of dysfunctional organelles (Uoselis et al., 2023). Parallel receptor-mediated pathways involving BCL2 interacting protein 3 (BNIP3), BCL2/adenovirus E1B 19kDa interacting protein 3-like (BNIP3L), FUN14 domain containing 1 (FUNDC1), and serine/threonine-protein phosphatase PGAM5 (mitochondrial - PGAM5) can bypass canonical ubiquitin signaling under specific stress conditions (Wang et al., 2026). Although LYC modulates multiple nodes within these pathways, it remains uncertain whether these effects arise from direct regulation of mitophagic machinery or from broader preservation of mitochondrial homeostasis through modulation of redox balance, inflammatory signaling, Ca^2+^ handling, and mitochondrial proteostasis. This distinction is central for determining whether LYC functions primarily as a regulator of mitochondrial quality control or as a broader redox-metabolic modulator whose effects on mitophagy are secondary to upstream mitochondrial stabilization.

**TABLE 5:** The effects of LYC on mitophagy.

Biological target	Experimental model	Major findings	References
ICR mice spleen	LYC at 5 mg/kg.day^-1^ during the administration of DEHP at 500–1000 mg/kg.day^-1^ (both orally administrated) for 28 days	Reduced mitochondrial vacuolization, chromatin marginalization, nuclear atrophy, and autophagosome formation; restored number of mitochondria per cell; attenuated the increase in the protein levels of SIRT1, SIRT3, SIRT7, and Mn-SOD; restored the protein levels of SIRT4 and SIRT6; reduced the protein levels of PGC-1α, OPA1, MFN2, MFN1, NRF1, DRP1, and TFAM; reduced the mRNA levels and protein levels of PINK1 and parkin; downregulated LC3-II Reduced the number of pro-inflammatory cells	Dai et al. (2021)
ICR mice thymus	LYC at 5 mg/kg.day^-1^ during the exposure to ATZ at 50–200 mg/kg.day^-1^ for 21 days (intragastrical administration for both compounds)	Reduced the levels of TNF-α and IL-6 and increased the IL-10 amounts in mice serum Decreased the levels of NF-κB, AIM2, ASC, caspase-1, and GSDMD- terminal Suppressed mitochondrial cristae reduction and ablation; downregulated LC3B-II and p62 protein levels; reduced PINK1 and parkin protein levels and mitochondrial localization; decreased FOXO1 and STAT3 protein levels Attenuated growth reduction of thymus	Zhu et al. (2022)
ICR mice kidney	LYC at 5 mg/kg.day^-1^ during the exposure to DEHP at 500–1000 mg/kg.day^-1^ for 28 days (both orally administrated through gavage)	Downregulated CXCL10, TNF-α, and IL-6 gene expression and protein levels Decreased XBP1-s and XBP1-u gene expression and protein levels Restored the expression of non-nuclear mitochondrial DNA, ND1, and COX1; preserved MMP and ATP levels; decreased PINK1, parkin, LC3B, and p62/SQSTM1 protein levels Downregulated cGAS and STING gene expression and protein levels Attenuated NF-κB activation; decreased bax, bak, and caspase-3 protein levels; diminished the number of apoptotic cells Improved morphological alterations	Li et al. (2023)
ICR mice kidney	LYC at 200 mg/kg (added to the diet) during exposure to potassium dichromate (K_2_Cr_2_O_7_) at 100 mg/kg (administrated through gavage) for 28 days	Suppressed mitochondrial vacuolar degeneration and cristae disruption; reduced the distance between mitochondria and endoplasmic reticulum Decreased H_2_O_2_ levels; attenuated lipid peroxidation; increased GPX and CAT enzyme activity; enhanced Nrf2, NQO1, CAT, GPX, Cu/Zn-SOD, GCLC, and GCLM mRNA levels; augmented Nrf2 protein levels Reduced DRP1 mRNA and protein levels; upregulated SIRT1 and PGC-1α protein levels; diminished DRP1, MFF, and FIS1 mRNA levels;	Zhang et al. (2026)
​	​	induced MFN1, MFN2, OPA1, SIRT1, PGC-1α, and TFAM gene expression; decreased PINK1, parkin, ATG5, ATG7, and p62 gene expression and protein levels; reduced LC3-I and LC3-II protein levels (leading to reduced LC3II/LC3I ratio) Alleviated vacuolar degeneration of renal tubular epithelial cells; attenuated structural abnormalities in glomerular epithelial cells	​
ICR mice heart	LYC at 5 mg/kg.day^-1^ during the exposure to DEHP at 500–1000 mg/kg.day^-1^ for 4 weeks (both orally administrated through gavage)	Downregulated CK, LDH, and α-HBDH Attenuated ultrastructural abnormalities (*i.e.*, enlarged mitochondria, broken mitochondrial cristae, and decreased number of mitochondria) Decreased H_2_O_2_ levels and the activity of GST and MPO; increased GSH levels and GPX activity Downregulated PINK1 and parkin gene expression and protein levels; reduced BNIP3, TOM20, TOM40, TOM70, and p62 gene expression Upregulated PGC-1α, SIRT1, and NRF1 gene expression and protein levels; stimulated SIRT3 gene expression; upregulated OPA1, MFN1, and MFN2 gene expression and protein levels Ameliorated heart histopathologic parameters	Shen et al. (2023)
ICR mice liver	LYC at 5 mg/kg.day^-1^ during the exposure to DEHP at 500–1000 mg/kg.day^-1^ for 4 weeks (both orally administrated through gavage)	Reduced hepatic ultrastructural damage (*i.e.*, mitochondrial autophagic vesicles, cytoplasmic lipid droplets, mitochondrial swelling, blurry mitochondrial membranes, mitochondrial volume density, and loss of mitochondrial membrane potential) Increased citrate synthase activity; upregulated COX1 and ND1; upregulated the expression and protein levels of SIRT1, NRF1, PGC-1α, SIRT3, TFAM, TFB1M and COXIV Reduced PINK1, parkin, and Beclin1 protein levels; diminished the expression of Fundc1, ATG5, ATG7, NDP52 and OPTN Downregulated HSF1, HSF2 and HSF4 gene expression; reduced CLPP, LONP1, ATF5, MRPP3, SIRT7 and YME1L1 gene expression; decreased HSP60, HSP70, HSP90, and HSF1 protein levels Attenuated hepatic histopathological abnormalities	Zhao et al. (2022)
C57 mice liver	LYC at 5 mg/kg.day^-1^ (BW, through gavage) during exposure to T-2 toxin at 1 mg/kg.day^-1^ (BW, through gavage) for 28 days	Decreased cytoplasmic vacuolization; diminished iron content, ROS production, and 4-HNE and MDA levels; enhanced nuclear Nrf2 levels; restored GPX4 enzyme activity and protein levels; augmented NQO1 and HO-1 protein levels Increased LC3, p62, PINK1, and parkin protein levels; knocking out of parkin (*Parkin* ^*−/−*^ animals) potentiated T-2-induced ferroptosis Increased SLC40A1 (ferroportin) protein levels; decreased FTH and FTL protein levels; inhibition of ferroptosis (by Ferrostatin-1) alleviated hepatotoxicity caused by T-2 toxin Improved hepatic function	Yang et al. (2026)
ICR mice testes	LYC at 5 mg/kg.day^-1^ during the exposure to DEHP (at 500–1000 mg/kg.day^-1^) for 28 days (both orally administrated through gavage)	Increased mitochondria volume density and MMP; upregulated PGC-1α, SIRT1, SIRT3, NRF1, TFAM, and COXIV gene expression and protein levels; enhanced MFN1, MFN2, and OPA1 gene expression and protein levels; decreased FIS1, DRP1, MFF, MIEF1, and MIEF2 gene expression Decreased PINK1, parkin, Beclin1, TOM20, TOM40, TOM70, FUNDC1, FOXO3, VDAC1, BNIP3, OPTN, ULK1, and ATG5 gene expression and protein levels; elevated p62 gene expression and protein levels Downregulated HSFs gene expression and protein levels Decreased CLPP, LOMP, MRPP3, SIRT7, HTRA2, and ATF5 gene expression and protein levels	Zhao et al. (2020)
BALB/c mice liver	LYC at 5 mg/kg.day^-1^ during the exposure to ATZ at 50–200 mg/kg.day^-1^ for 21 days (intragastrical administration for both compounds)	Augmented NAD^+^ and decreased NADH levels; enhanced both NADP^+^ and NADPH concentration; decreased mitochondrial vacuolation and cristae abnormalities; restored mitochondrial volume density Increased MFN1, MFN2, and OPA1 protein levels; reduced DRP1 protein levels; decreased Beclin1 and total and mitochondrial PINK1 and parkin protein levels; increased mitochondrial p62 protein levels; reduced mitophagy rate Reduced HSP60, CHOP, GRP75, CLPP, LONP1, and ATF5 protein levels (indicating attenuation of mitochondrial stress) Increased COX1 and ND1 protein levels; decreased mtDNA damage; restored PGC-1α, SIRT1, SIRT3, NRF1, TFAM, and TFB1M mRNA levels; upregulated PGC-1α, SIRT1, and TFAM protein levels; decreased SIRT3 protein levels	Yang et al. (2023)
Wistar rat testes	LYC at 25 mg/kg (orally administrated) during the exposure to IFO at 250 mg/kg (i.p. injected) for 60 days	Downregulated PINK1, parkin, LC3-I gene expression	Thoulfikar et al. (2025)
Chicken hepatocellular carcinoma LMH cell line	LYC at 1 µM during the exposure to FB1 at 50 µM for 24 h	Attenuated mitochondrial swelling, vacuolation, and cristae fragmentation; restored MMP Decreased caspase-7, caspase-3, and ZBP1 protein levels; decreased ROS production; downregulated GSDMD-terminal, caspase-1 and NLRP3 and phospho-RIPK1, phospho-RIPK3, and phospho-MLKL protein levels Attenuated apoptosis, pyroptosis, and necroptosis Upregulated PINK1, parkin, Beclin1, and LC3-II/I protein levels; downregulated TOM20 and p62 immunocontents Increased SIRT1 protein levels; decreased acetylated-FOXO1 protein levels; did not alter FOXO1 total protein levels; silencing of SIRT1 suppressed the effects induced by LYC on PINK1, parkin, Beclin1, LC3-II/I, TOM20, and p62; exerted SIRT1-dependent effects on PANoptosis	Wang et al. (2025)
Chicken hepatocellular carcinoma LMH cell line	LYC at 1 µM during the exposure to FB1 at 25 µM for 24 h	Downregulated p21, p16, p53, and γ-H2AX protein levels Upregulated ATG5, ATG7, Beclin1, and LC3-II/I and decreased LAMP2 and p62 protein levels Relieved the inhibition caused by FB1 on mitophagy (mito-Keima analyses) Upregulated SIRT3, FOXO3a, BNIP3L, and BNIP3 and downregulated TOM20 protein levels Attenuated loss of MMP, ROS production, and opening of the mPTP; silencing of SIRT3 abolished the effects induced by LYC on mitophagy, mitochondrial function, and senescence	Chang et al. (2025)
Rat renal tubular duct epithelial NRK52E cell line and C57BL/6 mice kidney	*In vitro* LYC at 10 µM during the exposure to AAI at 40 µM for 24 h *In vivo* LYC at 20 mg/kg.day^-1^ during exposure to AAI at 10 mg/kg.day^-1^ for 28 days (oral administration through gavage)	*In vitro* Upregulated PINK1, parkin, SQSTM1 protein levels; stimulated autophagosome formation; increased ATP levels; restored MMP; stimulated mitophagy by inhibiting Akt Downregulated the TGF-β/SMAD2/SMAD3 signaling pathway; attenuated epithelial-mesenchymal transition; effects dependent on mitophagy induction by LYC *In vivo* Alleviated mitochondrial swelling, vacuolation, and fragmentation; increased the number of autophagosomes; stimulated conversion of autophagosomes into autolysosomes; upregulated LC3-II, PINK1, parkin, and SQSTM1 protein levels Restored kidney size; attenuated infiltration of inflammatory cells and glomerular atrophy; recovered renal function; reduced collagen deposition; attenuated renal fibrosis	Wang et al. (2024c)
PC12 and SH-SY5Y cells and mice brain, substantia nigra, and striatum	*In vitro* Sequence-targeted nanodots (TPP-rHuHF-LYC; at 200 nM) administrated to PC12 cells challenged with MPP^+^ at 1 mM for 24 h *In vivo* TPP-rHuHF-LYC (intravenous administration) after exposure to MPTP (at 20 mg/kg.day^-1^) for 1 week (i.p. administration)	*In vitro* Attenuated Ca^2+^ overload; decreased total and mitochondrial ROS production; attenuated mPTP opening; restored ATP levels and NADH/NAD^+^ redox couple; restored OCR, ATP production, maximal respiration, spare capacity, and proton leak Increased PINK1 and parkin mRNA and protein levels; increased LC3-II/LC3-I ratio; augmented the formation of intracellular membrane structures; maintained mitochondrial branching degree; attenuated mitochondrial swelling and cristae ablation; enhanced free membrane around mitochondria Attenuated Rab10 levels and α-synuclein protein levels; increased TH protein levels Stimulated the nrf2/HO-1 axis; inhibition of autophagy (by using 3- 3-MA) suppressed the TPP-rHuHF-LYC-induced mitochondrial and neuronal protection *In vivo* Increased dopamine and acetylcholine levels; reduced acetylcholinesterase activity; stimulated SDH activity; augmented ATP levels; reduced NADH/NAD^+^ ratio; enhanced TH mRNA and protein levels; reduced α-synuclein mRNA and protein levels; attenuated GFAP and Iba-1 protein levels; enhanced PINK1 and parkin mRNA and protein levels; increased LC3-II/LC3-I ratio; increased Beclin1 and LAMP1 protein levels; decreased p62 protein levels; increased MFN2 and Bcl-xL protein levels; reduced FIS1, DRP1, VDAC1, and bax protein levels; reduced phosphorylated Akt and cleaved caspase-3 protein levels Attenuated motor impairment	Xia et al. (2023)
Porcine small intestinal epithelial IPEC-J2 cell line	LYC at 30 μg/mL during the exposure to DON at 148.16 ng/mL for 24 h	Decreased reactive oxygen species production and lipid peroxidation; suppressed mPTP opening; restored MMP; upregulated expression of OPA1, MFN1, and MFN2; downregulated the expression of MFF, MIEF1, FIS1, and DRP1; reduced the protein levels of DRP1; reduced PINK1 and parkin protein levels; decreased the expression of p62 and LC3 Attenuated loss of cell viability; reduced intestinal epithelial barrier disruption	Cai et al. (2023)
Porcine small intestinal epithelial IPEC-J2 cell line	LYC at 1 µM during exposure to DON at 1 µM for 24 h	Improved cell viability; reduced tight junction disruption Attenuated mitochondrial swelling and vacuolization; abrogated cristae disruption; suppressed loss of MMP; diminished mitochondrial ROS production Lowered ferrous ion levels; augmented GSH content; attenuated lipid peroxidation; decreased ACSL4 and TFRC protein levels; upregulated GPX4, SLC7A11, PCBP1, and FTH1 protein levels Downregulated PINK1, parkin, LC3-II/LC3-I, and PGAM5 protein levels; upregulated p62 and TOM20 protein levels; mitigated mitochondrial autophagic flux; PGAM5 overexpression abrogated the LYC-induced blockade on excessive mitophagy and on the effects associated with ferroptosis	Zheng et al. (2026)

In toxicant-induced mammalian models, particularly those involving DEHP, LYC consistently reduced PINK1/Parkin signaling, LC3-II accumulation, and expression of multiple autophagy-related proteins while simultaneously restoring mitochondrial morphology, ATP production, mitochondrial number, and mitochondrial biogenesis-related pathways (Zhao et al., 2020; Dai et al., 2021; Zhao et al., 2022; Li et al., 2023; Shen et al., 2023). These findings are commonly interpreted as suppression of excessive mitophagy; however, such interpretation is confounded by the absence of flux measurements. For example, decreased LC3-II levels may reflect reduced autophagosome formation, enhanced autophagosome clearance, or impaired autophagic initiation (Ding and Yin, 2012; Toh et al., 2013). Furthermore, reduced p62 levels may indicate enhanced degradation or decreased cargo recognition (Toh et al., 2013). Similarly, reduced PINK1/Parkin expression may arise from diminished mitochondrial depolarization rather than direct inhibition of mitophagic signaling (Wang et al., 2023c). Moreover, concurrent restoration of PGC-1α/TFAM signaling, mitochondrial fusion proteins (MFN1/2 and OPA1), mtDNA stability, and respiratory competence suggests that LYC primarily attenuated upstream mitochondrial injury, thereby reducing the requirement for compensatory mitochondrial turnover (Wang et al., 2026). In this context, modulation of SIRT1/sirtuin 3 (SIRT3) signaling, suppression of cGAS/STING-mediated inflammatory signaling, attenuation of UPR^mt^, and regulation of heat shock response systems further support the interpretation that LYC broadly stabilizes mitochondrial homeostasis rather than selectively targeting mitophagic machinery (Zhao et al., 2026). Notably, inhibition of cGAS/STING signaling may reflect reduced mtDNA instability and diminished mitochondrial danger-associated signaling secondary to preservation of mitochondrial integrity (Bai et al., 2017; Zhou et al., 2021). A similar interpretative limitation is evident in the study by Thoulfikar et al. (2025), performed in ifosfamide (IFO)-exposed Wistar rat testes, in which LYC altered mRNA expression levels of mitophagy-related markers, including PINK1, Parkin, and LC3-I. However, because neither mitochondrial function nor mitochondrial turnover was directly evaluated, the biological significance of these transcriptional changes remains unresolved. These observations reinforce a broader limitation across the field, namely the frequent reliance on isolated transcriptional or protein markers in the absence of dynamic assessment of mitochondrial quality control.

This interpretation was further supported in potassium dichromate (K_2_Cr_2_O_7_)-induced nephrotoxicity, where LYC simultaneously attenuated oxidative stress, restored mitochondrial dynamics, activated Nrf2-dependent antioxidant responses, and reduced PINK1/Parkin-, ATG5-, and ATG7-related signaling (Zhang et al., 2026). Notably, the concomitant induction of mitochondrial biogenesis pathways alongside reduced catabolic signaling raises an unresolved mechanistic question regarding how LYC coordinates the balance between mitochondrial renewal and degradation. Rather than directly suppressing mitophagy initiation, these findings suggest that LYC may shift mitochondrial quality control toward preservation of mitochondrial integrity and maintenance of bioenergetic competence.

A similar mechanistic complexity is evident in ATZ-induced thymic (Zhu et al., 2022) and hepatic (Yang et al., 2023) injury models, where LYC reduced mitochondrial PINK1/Parkin accumulation together with suppression of inflammasome activation, mitochondrial stress responses, and mtDNA instability. Given that mitochondrial damage and mtDNA release can activate both mitophagy and inflammasome pathways, the observed effects may reflect a common upstream event (namely, preservation of mitochondrial integrity) rather than direct cross-regulation between mitophagy and inflammation (Jing et al., 2020; Wang et al., 2026). The proposed STAT3/forkhead box protein O1 (FOXO1)/PINK1 axis provides a plausible transcriptional framework; however, without genetic or pharmacological manipulation of these components, it remains unclear whether LYC directly interferes with mitophagy initiation or indirectly reduces its activation by limiting mitochondrial stress signals. Thus, the apparent suppression of mitophagy may represent a reduced requirement for mitochondrial turnover, rather than inhibition of the process itself. Likewise, in the liver, LYC decreased markers of mitochondrial stress [heat shock protein 60 (HSP60), caseinolytic mitochondrial matrix peptidase proteolytic subunit (CLPP), Lon peptidase 1 (LONP1), activating transcription factor 5 (ATF5)], restored mitochondrial biogenesis pathways, and reduced mitochondrial turnover while preserving mtDNA integrity (Yang et al., 2023). However, inconsistencies in the regulation of specific proteins and the absence of functional assessment complicate interpretation of whether LYC truly suppresses mitophagy or instead promotes adaptive remodeling of mitochondrial quality control networks. The lack of pharmacological or genetic perturbation of mitochondrial biogenesis or mitophagy components further limits establishment of causal hierarchy between these processes.

In contrast, models characterized by severe accumulation of dysfunctional mitochondria reveal a distinct pattern in which LYC appears to facilitate selective mitochondrial clearance. In fumonisin B1 (FB1)-exposed LMH hepatocarcinoma cells, LYC activated PINK1/Parkin signaling, increased LC3-II formation, reduced translocase of outer mitochondrial membrane 20 (TOM20) and p62 accumulation, and restored mitochondrial function in a SIRT1-dependent manner (Wang et al., 2025). Silencing of SIRT1 partially abrogated these effects, providing important causal support for involvement of mitochondrial turnover pathways. Nevertheless, the mechanistic hierarchy linking SIRT1 activation to PINK1 stabilization and Parkin recruitment remains unresolved. SIRT1 may act indirectly through deacetylation of transcription factors (e.g., FOXO1) or modulation of mitochondrial biogenesis pathways, but direct effects on mitophagy machinery have not been demonstrated (Wang et al., 2023a; Guan et al., 2025). Similarly, Chang et al. (2025) demonstrated that LYC activates the SIRT3/Forkhead Box O3a (FOXO3a)/BNIP3L axis while restoring mitochondrial turnover assessed using mito-Keima assays, thereby providing one of the most mechanistically rigorous demonstrations of LYC-induced mitophagy currently available. Importantly, SIRT3 silencing abolished the effects of LYC on mitochondrial function, mitophagy, and cellular senescence, strongly suggesting that receptor-mediated mitophagy contributes directly to cytoprotection in this context. A related pro-mitophagic profile was observed in T-2 toxin-induced hepatotoxicity, where LYC increased PINK1, Parkin, LC3, and p62 levels while attenuating ferroptosis and restoring glutathione peroxidase 4 (GPX4) activity through activation of the Nrf2 axis (Yang et al., 2026). The use of Parkin-deficient animals further strengthened the mechanistic association between mitochondrial turnover and hepatoprotection, as disruption of Parkin signaling exacerbated ferroptosis and liver injury. However, because mitophagy was not selectively inhibited in LYC-treated animals, it remains uncertain whether enhanced mitochondrial turnover directly mediated ferroptosis suppression or whether both phenomena emerged from broader restoration of redox homeostasis. Moreover, the proposed interaction of LYC with Keap1/Nrf2 complexes, inferred from molecular docking analyses, still requires biochemical validation under physiologically relevant conditions. Collectively, these studies suggest that LYC may facilitate removal of irreversibly damaged mitochondrial subpopulations in highly oxidative environments; however, the extent to which this reflects direct activation of mitophagic machinery versus restoration of mitochondrial signaling competence remains unresolved.

Additional complexity emerges in intestinal epithelial models challenged with DON. Cai et al. (2023) observed that LYC reduced PINK1/Parkin signaling together with improvements in mitochondrial dynamics and epithelial barrier integrity, again suggesting reduced mitochondrial turnover secondary to preservation of mitochondrial function. In contrast, Zheng et al. (2026) provided substantially stronger mechanistic evidence by combining mito-Keima analyses with PGAM5 overexpression approaches. Their data demonstrated that LYC suppresses excessive mitochondrial turnover through modulation of PGAM5-mediated signaling, thereby attenuating ferroptosis and restoring epithelial barrier integrity. The observation that PGAM5 overexpression abolished the cytoprotective effects of LYC suggests a direct mechanistic relationship between mitochondrial turnover and ferroptosis control. Moreover, molecular docking analyses indicating potential interaction between LYC and PGAM5 raise the possibility that LYC may directly modulate mitophagy-related proteins under specific pathological conditions. Nevertheless, whether these interactions occur at physiologically relevant intracellular concentrations remains unresolved.

Renal injury models induced by aristolochic acid I (AAI) further illustrate the adaptive nature of LYC-mediated mitochondrial remodeling. Wang et al. (2024c) demonstrated that LYC enhanced autophagosome formation, stimulated autophagosome-to-autolysosome conversion, activated PINK1/Parkin signaling, and restored ATP production and MMP while suppressing PI3K/Akt/mechanistic target of rapamycin (mTOR) signaling. Unlike DEHP-associated models, where LYC reduced mitophagy-related markers, these findings suggest restoration of mitochondrial turnover capacity in severely damaged mitochondria. While inhibition of mTOR is a canonical trigger of autophagy, it is not specific to mitophagy, raising the possibility that LYC induced a broader autophagic response rather than selective mitochondrial clearance (Deleyto-Seldas and Efeyan, 2021). The simultaneous improvement of mitochondrial bioenergetics alongside activation of mitochondrial clearance pathways challenges the classical paradigm that mitophagy is merely a downstream consequence of mitochondrial dysfunction. Instead, these findings support the possibility that LYC facilitates selective removal of irreversibly damaged mitochondrial subpopulations while preserving functionally competent organelles. Nonetheless, without flux-specific assays or selective inhibition of mitophagy components, it remains unclear whether mitochondrial turnover is increased, normalized, or redistributed across subpopulations of mitochondria.

More mechanistically resolved insights emerge from mitochondria-targeted formulations, such as triphenylphosphonium (TPP)-conjugated LYC nanodots (Xia et al., 2023). These formulations promoted mitochondrial accumulation, enhanced mitophagic signaling, restored respiratory function, normalized oxygen consumption (OCR) and ATP production, and improved neuronal survival in Parkinsonian models. Importantly, pharmacological inhibition of autophagy abolished the protective effects induced by mitochondria-targeted LYC, providing strong evidence that mitochondrial turnover contributed causally to mitochondrial and neuronal rescue. These findings also highlight a critical but often overlooked variable across the broader literature: effective mitochondrial delivery. The superior efficacy of targeted nanodots suggests that free LYC may exert predominantly indirect effects, whereas mitochondrial accumulation enables direct modulation of organelle-specific pathways. Furthermore, modulation of leucine-rich repeat kinase 2 (LRRK2)/Ras-related protein Rab-10 (Rab10) signaling and α-synuclein clearance indicates that LYC-induced mitophagy may intersect with broader proteostatic and neurodegenerative pathways (Park et al., 2025).

Taking together, current evidence supports a model in which LYC dynamically adjusts mitochondrial turnover according to the severity and nature of mitochondrial stress. Rather than functioning as a simple activator or inhibitor, LYC appears to recalibrate the threshold for mitophagy initiation, suppressing excessive mitochondrial degradation under conditions of moderate injury while promoting selective clearance when dysfunctional mitochondria accumulate beyond repair capacity. Emerging evidence further suggests that LYC-mediated modulation of mitophagy intersects with broader mitochondrial stress adaptation programs, including ferroptosis suppression, inflammasome regulation, and Nrf2-dependent redox signaling, indicating that mitochondrial turnover is coordinated within an integrated cytoprotective network rather than regulated as an isolated process. In this context, mitophagy may represent not only a mechanism of mitochondrial quality control, but also a central node linking redox homeostasis, metabolic adaptation, and cell fate determination.

However, this framework remains largely conceptual because the mechanistic hierarchy linking redox modulation, inflammatory signaling, mitochondrial proteostasis, ferroptosis-related pathways, and core mitophagic machinery has not been definitively established. A major limitation across the field remains the predominance of marker-based evidence in the absence of rigorous flux analyses, causal genetic manipulation, and quantitative assessment of mitochondrial function. Consequently, it remains unresolved whether LYC directly regulates mitophagic machinery through effects on protein activation, localization, or organelle-specific signaling, or whether observed changes in mitochondrial turnover emerge secondarily from broader improvements in mitochondrial integrity and cellular homeostasis. This distinction is particularly relevant given recent evidence suggesting potential interactions of LYC with regulatory proteins associated with mitophagy and redox signaling, although these observations still require biochemical and pharmacological validation under physiologically relevant conditions.

Considering future directions, standardized flux quantification using mito-Keima or tandem mito-mCherry-GFP reporters combined with lysosomal inhibitors should be adopted broadly to discriminate increased autophagosome formation from successful mitophagic degradation (Katayama et al., 2011; Sun et al., 2015; McWilliams et al., 2016). Second, rigorous causal testing using genetic loss-of-function models for PINK1, Parkin, BNIP3/BNIP3L, and sirtuins (SIRT1/SIRT3) is required to determine whether LYC-induced benefits are mitophagy-dependent. Third, comparative pharmacokinetic and subcellular distribution studies are necessary to define how formulation and mitochondrial targeting affect efficacy and signaling bias (Liu et al., 2006). Fourth, integrated metabolic profiling (OCR, ATP, OXPHOS complexes activities) must be paired with mitophagy assays to link molecular modulation with functional recovery. Fifth, systematic cross-tissue comparisons (tumor vs. neuronal vs. renal vs. immune cells) under standardized stressors will clarify whether cellular metabolic context predicts the directionality of the effects promoted by LYC. Finally, exploration of the interaction of LYC with inflammation and immune signaling may reveal indirect routes by which LYC modulates mitophagy (Guilbaud et al., 2024).

## 3 Translational perspective

Despite compelling experimental evidence supporting the mitochondrial activity of LYC, its clinical translation remains limited by a convergence of pharmacokinetic constraints, structural instability, and incomplete mechanistic resolution. A critical limitation is the disparity between concentrations used *in vitro* and those achievable in humans, where circulating levels are typically low and highly variable (within the low micromolar to nanomolar range) (Holzapfel et al., 2013; Shafe et al., 2024). This is compounded by poor aqueous solubility, susceptibility to oxidative degradation and isomerization, and strong dependence on dietary context for absorption, collectively resulting in unpredictable systemic exposure and uncertain mitochondrial target engagement (Clinton, 1998; Shafe et al., 2024). In that context, although selective tissue accumulation and preliminary evidence of mitochondrial enrichment have been reported, the extent to which LYC reaches mitochondria at functionally effective concentrations remains insufficiently defined.

At a mechanistic level, the translational trajectory of LYC is further complicated by ambiguity regarding its primary mode of action. Although LYC influences mitochondrial parameters (including redox balance, membrane potential, and apoptotic signaling) these effects are often intertwined with upstream regulatory networks such as Nrf2 and AMPK, as discussed here. Dissecting whether LYC acts as a direct mitochondrial effector or as a systems-level modulator of cellular stress responses is essential for defining its therapeutic positioning. This distinction is particularly relevant given its context-dependent effects, which vary across metabolic and oncological conditions with divergent mitochondrial phenotypes.

Importantly, the biological effects of LYC appear to be highly context-dependent, varying across disease states characterized by distinct mitochondrial phenotypes. In metabolically stressed or degenerative conditions, LYC may restore mitochondrial function and redox balance, whereas in cancer, it may differentially modulate bioenergetics and apoptotic sensitivity. This context specificity further complicates its translational application and underscores the need for disease-tailored evaluation.

Advancing LYC toward clinical applicability will require a coordinated, mechanism-informed optimization strategy. Prioritization of cis-isomer-enriched formulations represents a rational starting point, as these species exhibit enhanced bioavailability compared to the all-trans isomer predominant in native dietary sources (Boileau et al., 2002; Unlu et al., 2007). In parallel, formulation strategies must address both solubility and chemical stability, preserving molecular integrity while improving absorption kinetics. In this regard, nanotechnology-based delivery systems offer a particularly promising avenue (Falsafi et al., 2022). Nanoformulations, including lipid-based carriers and mitochondria-targeted constructs incorporating lipophilic cations, may enhance tissue distribution, promote subcellular accumulation, and enable more precise interrogation of the direct mitochondrial interactions of LYC.

Complementary to formulation advances, synergistic intervention strategies may further strengthen translational potential. Co-administration with other antioxidants could stabilize LYC and amplify its redox-modulating effects through cooperative mechanisms, while whole-food-derived formulations (such as tomato-based matrices) may improve bioavailability and more faithfully reproduce the complex biochemical environment associated with dietary intake (Shi and Le Manguer, 2000). These approaches may mitigate limitations inherent to isolated compound administration and enhance physiological relevance.

From a clinical development perspective, future progress will depend on rigorously designed, biomarker-driven trials that integrate pharmacokinetic-pharmacodynamic modeling with organelle-specific functional endpoints. Quantitative assessment of mitochondrial exposure, alongside validated biomarkers of mitochondrial function and redox status, will be essential to establish causal links between LYC bioavailability and therapeutic outcomes. Stratification of patient populations based on metabolic and mitochondrial phenotypes should further refine response prediction and optimize trial design. Ultimately, resolving whether LYC functions as a mitochondria-directed therapeutic or as a broader regulator of cellular homeostasis will be determinative for its positioning within precision medicine frameworks.

## 4 Comparison with mitochondria-directed pharmacological agents

A critical distinction in the translational positioning of LYC emerges when compared with mitochondria-targeted compounds such as MitoQ, SkQ, and Szeto-Schiller peptides (e.g. SS-31, Elamipretide). Unlike these agents, which are specifically engineered to accumulate within mitochondria through defined targeting strategies [either via membrane potential-driven uptake (TPP-conjugated antioxidants) or cardiolipin-binding motifs (Szeto-Schiller-peptides)] LYC lacks a dedicated mitochondrial delivery mechanism (James et al., 2007; Skulachev et al., 2009; Szeto et al., 2011; Szeto and Schiller, 2011). Consequently, while MitoQ and SkQ exert localized antioxidant effects directly within the mitochondrial matrix, and SS-peptides stabilize inner membrane architecture and ETC organization, LYC appears to modulate mitochondrial function through a combination of indirect redox regulation and broader signaling pathways, including Nrf2, AMPK, and inflammatory cascades. This fundamental difference suggests that LYC should be considered a non-targeted, systems-level modulator of mitochondrial homeostasis, rather than a mitochondria-directed pharmacological agent, with important implications for its pharmacokinetic–pharmacodynamic profile and translational applicability.

## 5 Conclusion

Collectively, the preclinical literature supports that LYC is a multi-modal mitochondrial protector that modulates redox balance, stabilizes OXPHOS, limits mitochondria-mediated apoptosis, and influences mitochondrial quality control. Yet, inconsistent reporting, limited causal experiments, variable dosing/pharmacokinetics data, and a paucity of rigorous analyses of mitochondrial dynamics constrain mechanistic certainty and translational advance. Addressing the specific experimental gaps above will be essential to determine whether LYC (or mitochondria-targeted LYC formulations) can be validated as therapeutics that restore mitochondrial homeostasis in human disease.

LYC consistently protects mitochondrial structure and function in diverse preclinical models. Across neuronal, cardiac, hepatic and reproductive systems, LYC reduces mitochondrial ROS and lipid peroxidation, preserves MMP, and rescues activities of respiratory complexes and ATP content after toxic or inflammatory insults. These effects are reported both *in vitro* and *in vivo* and have been reproduced using different delivery strategies including mitochondria-targeted nanodots that improve neural and mitochondrial bioavailability.

Mechanistically, multiple studies implicate activation of canonical antioxidant and metabolic regulators (Nrf2/HO-1, SIRT1/3, PGC-1α, AMPK) and suppression of inflammatory signaling [NF-κB, Toll-like receptor 4 (TLR4)]. These upstream changes correlate with downstream stabilization of OXPHOS components, reduced opening of the mPTP, lower cytochrome c release, and reduced caspase activation, together supporting a mitochondria-centered cytoprotective program. However, the majority of reports are correlative: few employ loss- or gain-of-function approaches (*e.g.*, genetic knockdown of PINK1/Parkin, pharmacologic blockade of SIRT1 or Nrf2) to test causality. As a result, attribution of protection to specific signaling axes remains provisional.

Mitochondrial quality control (mitophagy and biogenesis) appears to be a key target of LYC, but with heterogeneous outcomes. Several studies show induction of canonical mitophagy markers (PINK1/Parkin, LC3-II) and functional mitophagy that parallels organ protection; others report decreased mitophagy markers together with increased biogenesis markers (PGC-1α, TFAM), reflecting model-dependent differences or time- and dose-dependent responses. Notably, many studies rely on expression/activity readouts without demonstrating that mitophagy or biogenesis is necessary for the observed benefits (no rescue/ablation experiments).

Mitochondrial dynamics (fusion/fission) remain underexplored. A few reports note modulation of MFN1/2 and OPA1 or reductions in DRP1, but systematic, quantitative analyses of network morphology, dynamics (live-cell imaging), or functional consequences are scarce. Given the central role of dynamics in quality control and bioenergetics, this is a critical gap.

## Glossary

α-KGDH: α-ketoglutarate dehydrogenase
α-HBDH: α-hydroxybutyrate dehydrogenase
γ-H2AX: phosphorylated histone H2AX
3-MA: 3-methyladenosine
3-NP: 3-nitropropionic acid
4-HNE: 4-hydroxynonenal
Aβ: amyloid-β
**Aβ**_**1-42**_: amyloid-β peptide_1-42_
AAI: aristolochic acid I
ACC1: acetyl-CoA carboxylase 1
ACOX1: acyl-coenzyme A oxidase 1
ACSL4: acyl-CoA synthetase long chain family member 4
ADH: aldehyde dehydrogenase
**AFB**_**1**_: aflatoxin B1
AIF: apoptosis-inducing factor
AIM2: absent in melanoma 2
ALT: alanine aminotransferase
AMPK: AMP-activated protein kinase
Ang-2: angiopoietin-2
AOPP: advanced oxidation proteins products
APAF1: apoptotic protease activating factor-1
ASC: apoptosis-associated speck-like protein containing a CAR
AST: aspartate aminotransferase
ATF4: activating transcription factor 4
ATF5: activating transcription factor 5
ATF6: activating transcription factor 6
ATG5: autophagy-related protein 5
ATG7: autophagy-related protein 7
ATOX1: antioxidant 1 copper chaperone
ATP: adenosine triphosphate
ATP7A: ATPase copper transporting α
ATP7B: ATPase copper transporting β
ATPef08: F-type H^+^-transporting ATPase subunit 8
ATZ: atrazine
Bak: Bcl-2 homologous antagonist/killer
Bax: BCL2-associated X protein
Bcl-2: B-cell lymphoma 2
Bcl-xL: B-cell lymphoma-extra large
BDNF: brain-derived neurotrophic factor
BNIP3: BCL2 interacting protein 3
BNIP3L: BCL2/adenovirus E1B 19kDa interacting protein 3-like
CA1: Cornu Ammonis 1
CA3: Cornu Ammonis 3
CAT: catalase
CD31: platelet endothelial cell adhesion molecule-1
CD36: fatty acid translocase
CDKN1A: cyclin-dependent kinase inhibitor 1A
CDKN2A: cyclin-dependent kinase inhibitor 2A
cGAS: cyclic GMP-AMP synthase
CHOP: C/EBP homologous protein
CK: creatine kinase
CLPP: caseinolytic mitochondrial matrix peptidase proteolytic subunit
COX-2: cyclooxygenase-2
COX1: cytochrome c oxidase subunit 1
COX2: cytochrome c oxidase subunit II
COXIV: cytochrome c oxidase subunit 4
COX5a: cytochrome c oxidase subunit 5A
COX5B: cytochrome c oxidase subunit 5B, mitochondrial
COX7A1: cytochrome c oxidase subunit 7A1, mitochondrial
COX8B: cytochrome c oxidase subunit 8B, mitochondrial
COX17: cytochrome c oxidase copper chaperone
CPT-I: carnitine palmitoyltransferase-I
CTR1: copper transporter receptor
CXCL10: C-X-C motif chemokine ligand 10
CYCS: cytochrome c, somatic
CYP2E1: cytochrome P450 family 2 subfamily E member 1
D-gal: D-galactose
D-GalN: D-galactosamine
DBP: di-n-butyl phthalate
DEHP: di-(2-ethylhexyl) phthalate
DG: dentate gyrus
DLAT: dihydrolipoamide S-acetyltransferase
DON: deoxynivalenol
DRP1: dynamin-related protein 1
DT: dityrosine
E2F8: E2F transcription factor 8
eIF2α: eukaryotic initiation factor 2α
eNOS: endothelial nitric oxide synthase
ER: endoplasmic reticulum
ERK: extracellular signal-regulated kinase
ETC: electron transport chain
eWAT: epididymal white adipose tissue
FABP3: fatty acid binding protein 3
FB1: fumonisin B1
FDX1: ferredoxin 1
FFA: free fatty acid
FGF21: fibroblast growth factor 21
FIS1: mitochondrial fission 1 protein
FOXO1: forkhead box protein O1
FOXO3: forkhead box protein O3
FOXO3a: Forkhead Box O3a
FTH: ferritin heavy chain
FTH1: ferritin heavy chain 1
FTL: ferritin light chain
FUNDC1: FUN14 domain containing 1
GFAP: glial fibrillary acidic protein
GLUT1: glucose transporter 1
GLUT2: glucose transporter 2
GLUT3: glucose transporter 3
GLUT4: glucose transporter 4
GPX: glutathione peroxidase
GPX4: glutathione peroxidase 4
GR: glutathione reductase
GRP75: heat shock protein family A member 9
GRP78: 78 kDa glucose-regulated protein
**GSDMD**-terminal: gasdermin D N-terminal domain
GSH: glutathione
GSK3β: glycogen synthase kinase 3β
GSSG: oxidized glutathione
GST: glutathione-*S*-transferase
H/R: hypoxia/reoxygenation
HO-1: heme oxygenase-1
HSF: heat shock transcription factor
HSF1: heat shock transcription factor 1
HSF2: heat shock transcription factor 2
HSP60: heat shock protein 60
HSP70: heat shock protein 70
HSP90: heat shock protein 90
HTRA2: HtrA serine peptidase 2
I/R: ischemia/reperfusion
Iba-1: Ionized calcium-binding adapter molecule 1
iBAT: intrascapular brown adipose tissue
IDH: isocitrate dehydrogenase
IFN-γ: interferon-γ
IFO: ifosfamide
IκB: inhibitor of κB
IL-1β: interleukin-1β
IL-6: interleukin-6
IL-8: interleukin-8
IL-18: interleukin-18
iNOS: inducible nitric oxide synthase
IP3R1: inositol 1,4,5-trisphosphate receptor type 1
IRE1: inositol-requiring enzyme 1
IRS-1: insulin receptor substrate-1
JAK: Janus kinase
JAK2: Janus kinase 2
JNK: c-Jun N-terminal kinase
Keap1: kelch-like ECH-associated protein 1
LAMP1: lysosomal-associated membrane protein 1
LAMP2: lysosomal-associated membrane protein 2
LC3: microtubule-associated protein 1 light chain 3
LCAD: long chain acyl-CoA dehydrogenase
LDH: lactate dehydrogenase
LDL: low density lipoprotein
LIAS: lipoic acid synthetase
LONP1: Lon peptidase 1
LPS: lipopolysaccharide
LRRK2: leucine-rich repeat kinase 2
LYC: lycopene
MAM: mitochondria-associated membranes
MDA: malondialdehyde
MDH: malate dehydrogenase
MFF: mitochondrial fission factor
MFN1: mitofusin 1
MFN2: mitofusin 2
MIEF1: mitochondrial elongation factor 1
MIEF2: mitochondrial elongation factor 2
MLKL: mixed lineage kinase domain-like pseudokinase
MMP: mitochondrial membrane potential
MMP3: matrix metalloproteinase 3
Mn-SOD: manganese-dependent superoxide dismutase
MPO: myeloperoxidase
mPTP: mitochondrial permeability transition pore
MPTP: 1-methyl-4-phenyl-1,2,3,6-tetrahydropyridine
MRPP3: mitochondrial ribonuclease P catalytic subunit
mtDNA: mitochondrial DNA
mTOR: mechanistic target of rapamycin
NAC: N-acetylcysteine
**NAD** ^**+**^: nicotinamide adenine dinucleotide, oxidized form
NADH: nicotinamide adenine dinucleotide, reduced form
NADPH: nicotinamide adenine dinucleotide phosphate, reduced form
ND1: NADH-ubiquinone oxidoreductase chain 1
ND2: NADH dehydrogenase subunit 2
ND3: NADH dehydrogenase subunit 3
ND5: NADH-ubiquinone oxidoreductase chain 5
ND6: NADH:ubiquinone oxidoreductase core subunit 6
NDP52: nuclear domain 10 protein 52
NDUFS: NADH-ubiquinone oxidoreductase core subunit
NDUFS1: NADH-ubiquinone oxidoreductase 75 kDa subunit, mitochondrial
NDUFS2: NADH dehydrogenase [ubiquinone] iron-sulfur protein 2
NDUFS3: NADH dehydrogenase [ubiquinone] iron-sulfur protein 3, mitochondrial
NF-κB: nuclear factor-κB
NGF: nerve growth factor
NQO1: NAD(P)H quinone dehydrogenase 1
Nrf2: nuclear factor erythroid 2-related factor 2
NRF1: nuclear respiratory factor 1
NRF2: nuclear respiratory factor 2
NT3: neurotrophin-3
NT4: neurotrophin-4
OCR: oxygen consumption rate
OMA1: OMA1 zinc metallopeptidase
OPTN: optineurin
OPA1: optic atrophy 1
OXPHOS: oxidative phosphorylation
p62/SQSTM1: sequestosome 1
PCBP1: poly (rC) binding protein 1
PDGFR-β: platelet-derived growth factor receptor-β
PERK: protein kinase RNA-like endoplasmic reticulum kinase
PGAM5: serine/threonine-protein phosphatase PGAM5, mitochondrial
PGC-1α: peroxisome proliferator-activated receptor gamma coactivator 1-α
PI3K: phosphoinositide 3-kinase
PINK1: PTEN-induced putative kinase 1
PPARα: peroxisome proliferator-activated receptor-α
PPARγ: peroxisome proliferator-activated receptor-γ
PRDM16: PR domain zinc finger protein 16
PSD-95: postsynaptic density proteins 95
PTP1B: protein tyrosine phosphatase 1B
PTZ: pentylenetetrazol
Rab10: Ras-related protein Rab-10
RIPK1: receptor-interacting serine/threonine-protein kinase 1
RIPK3: receptor-interacting serine/threonine-protein kinase 3
RNS: reactive nitrogen species
ROS: reactive oxygen species
SASP: senescence-associated secretory phenotype
SCI: spinal cord injury
SDH: succinate dehydrogenase
SDHA: succinate dehydrogenase complex flavoprotein subunit A
SERCA2: sarcoplasmic/endoplasmic reticulum calcium ATPase 2
SIRT1: sirtuin 1
SIRT3: sirtuin 3
SIRT4: sirtuin 4
SIRT 6: sirtuin 6
SIRT7: sirtuin 7
SLC7A11: solute carrier family 7 member 11
SLC40A1: solute carrier family 40 member 1
SMZ: sulfamethoxazole
SNAP-25: synaptosomal-associated protein 25
SOD: superoxide dismutase
STAT3: signal transducer and activator of transcription 3
STING: stimulator of interferon genes
SYP: synaptophysin
*t*-BHP: *tert*-butyl hydroperoxide
TCA: tricarboxylic acid
TFAM: mitochondrial transcription factor A
TFB1M: mitochondrial transcription factor B1
TFRC: transferrin receptor
TH: tyrosine hydroxylase
TLR4: Toll-like receptor 4
TMT: trimethyltin
TNF-α: tumor necrosis factor-α
TOM20: translocase of outer mitochondrial membrane 20
TOM40: translocase of outer mitochondrial membrane 40
TOM70: translocase of outer mitochondrial membrane 70
TPP: triphenylphosphonium
UCP1: uncoupling protein 1
UCP2: uncoupling protein 2
UCP3: uncoupling protein 3
ULK1/2: unc-51 like autophagy activating kinase 1/2
**UPR** ^**ER**^: reticular unfolded protein response
**UPR** ^**mt**^: mitochondrial unfolded protein response
UQCRFS1: ubiquinol-cytochrome c reductase, Rieske iron-sulfur polypeptide 1
VAMP: synaptobrevin
VDAC1: voltage-dependent anion channel 1
VMP1: vacuole membrane protein 1
XBP1-s: spliced X-box binding protein 1
XBP1-u: unspliced X-box binding protein 1
YME1L1: ATP-dependent zinc metalloprotease YME1L1
YTHDF1: YTH N^6^-methyladenosine RNA binding protein 1
ZBP1: Z-DNA binding protein 1
ZEN: zearalenone
